# Impact of the national hepatitis B immunization program in China: a modeling study

**DOI:** 10.1186/s40249-022-01032-5

**Published:** 2022-10-11

**Authors:** Zhixi Liu, Mengying Li, David W. Hutton, Abram L. Wagner, Ye Yao, Wenlong Zhu, Lingsheng Cao, Shenglan Tang, Jinhua Pan, Yesheng Wang, Qi Zhao, Hong Ren, Ying Wang, Weibing Wang

**Affiliations:** 1 School of Public Health, Shanghai Institute of Infectious Disease and Biosecurity, Fudan University, Shanghai, China; 2 Department of Social Medicine, School of Public Health, Fudan University, Shanghai, China; 3 School of Public Health, University of Michigan, Ann Arbor, MI USA; 4 Department of Biostatics, School of Public Health, Fudan University, Shanghai, 200032 China; 5 Chinese Center for Disease Control and Prevention, National Immunization Program, Beijing, China; 6 Duke Global Health Institute, Duke University, Durham, NC USA; 7 Shanghai Municipal Center for Disease Control and Prevention, Shanghai, China

**Keywords:** Hepatitis B, National immunization program, Prevalence, Economic analysis, Susceptible-Exposed-Infectious-Recovered modeling

## Abstract

**Background:**

Elimination of hepatitis B virus (HBV) is a striking challenge for countries with high or moderate disease burden. Therefore, using China as a practical case to share experiences for similar countries may accelerate the achievement of the WHO 2030 target of 90% reduction in HBV-related incidence. We aim to evaluate the impact of national HBV immunization strategies in China; and the feasibility to achieve WHO 2030 targets under different scenarios.

**Methods:**

We constructed an expanded Susceptible-Exposed-Infectious-Recovered (SEIR) model and decision tree-Markov model to estimate the epidemic of HBV in China, assess the feasibility of 2030 Elimination Goals through the projections and conduct the economic analysis. Least square method was used to calibrate the expanded SEIR model by yearly data of laboratory-confirmed HBV cases from 1990 to 2018. Two models were separately used to evaluate the impact and cost-effectiveness of HBV vaccine by comparing prevalence of chronic HBV infections, quality-adjusted life-years (QALYs), incremental cost effectiveness ratio and benefit–cost ratio (BCR) under various intervention options, providing a basis for exploring new containment strategies.

**Results:**

Between 1990 and 2020, the number of chronic HBV infections decreased by 33.9%. The current status quo would lead to 55.73 million infections (3.95% prevalence) in 2030, compared to 90.63 million (6.42% prevalence) of the “Without the NIP” scenario (NIP: National Immunization Program), 114.78 million (8.13% prevalence) without any interventions. The prevention of mother to child transmission (PMTCT) strategy showed a net benefit as 12,283.50 dollars per person, with BCR as 12.66, which is higher than that of universal vaccination at 9.49. Compared with no screening and no vaccination, the PMTCT strategy could save 7726.03 dollars for each QALY increase.

**Conclusions:**

Our findings proved the HBV vaccination has demonstrated a substantial positive impact on controlling the epidemic of HBV in terms of effectiveness and economy after about 30 years of implementation of the national hepatitis B immunization program which also provided containment experience for high or medium burden countries. As for China, the next step should focus on exploring strategies to improve diagnosis and treatment coverage to reduce the burden of HBV-related deaths and ultimately eliminate HBV.

**Supplementary Information:**

The online version contains supplementary material available at 10.1186/s40249-022-01032-5.

## Background

Globally, hepatitis B is a major threat to public health due to its high transmissibility and the high incidence of chronic persistent and adverse outcomes [1, 2]. About 296 million people were living with chronic hepatitis B infection, which caused around 820,000 deaths globally in 2019 [3, 4]. However, even though HBV is among the top four global infectious diseases, in line with HIV, malaria and tuberculosis [5], its control is neglected and it isn’t a political priority in many countries [6].

Given the current burden of HBV [7], World Health Organization (WHO) developed a global plan for Hepatitis B elimination by 2030. This plan has been designed to increase attention and maintain momentum towards the control and elimination of hepatitis B among WHO Member States [8]. The 2030 elimination plan includes goals to increase pediatric and maternal immunization, and to improve screening and treatment, which, if achieved, would reduce annual incidence from hepatitis B by 90% (equivalent to 0.1% prevalence of HBsAg [Hepatitis B surface antigen] among children [9]) and achieve vaccination coverage of 90% by 2030 [10]. To fulfill these targets, it’s crucial for low- and middle-income countries to formulate appropriate strategies based on the local environment and epidemiological circumstance. These policies also need public investment and funding [11, 12].

Based on WHO criteria, China has been classed as a medium–high Hepatitis B endemic area [13, 14], with 70 million HBsAg positive infections [15] (nearly account for one third infections of global burden of HBV [16]), and about 28 million chronic hepatitis B (CHB) cases among them. China has instituted a variety of strategies and programs over time to control hepatitis B. In 1992, HBV vaccine was included in the national immunization program (self-paying for the fee of HBV vaccine and service). Since 2002, all newborns of must complete the vaccination without the fee of HBV vaccine [17]. In 2015, HBV immunoglobulin was injected into newborns within 24 h after birth based on the HBV vaccination, and hepatitis B screening was provided free of charge to all pregnant women.

For China, which accounts for approximately 30% of the global HBV burden, evaluating its containment course will not only accelerate the elimination of hepatitis B, but provide valuable insights for similar countries. We aim to quantify the impact and cost-effectiveness of immunization strategies compared with different counterfactual scenarios; and models can estimate if China will make the 2030 Elimination Plan goals, and, if not, what strategies could be implemented.

## Methods

### Data collection

In China, all laboratory-confirmed CHB cases (chronic HBV infections with chronic inflammatory liver disease) are required to be reported to the Chinese Center for Disease Control and Prevention (CDC) in the National Notifiable Disease Reporting System (NNDRS). The yearly reported number of CHB cases for 1990–2018 was extracted from National Population and Health Science Data Sharing Platform (https://www.phsciencedata.cn/Share/en/index.jsp) of the China CDC. Moreover, the sources of the HBV vaccination database (including infant and timely birth-dose vaccination rate) we established were: (1) data of the 1990–2003 HBV vaccination rates were from a national vaccination rate survey based on a multi-stage random sample conducted by Liang et al. [18]; (2) the 2004–2018 HBV vaccination rates were from WHO and the United Nations International Children's Emergency Fund (UNICEF) database based on national vaccination progress reports (http://apps.who.int/gho/data/node.main.A824?lang=en). Information on the total population size, birth rates and mortality rates were obtained from the statistical yearbook of the National Bureau of Statistics.

### Model conceptions

We developed a dynamic model and decision tree-Markov model to evaluate the impact, cost-effectiveness and returns of a 30-year HBV immunization program (involving multiple immunization strategies) in China. The framework of these two models is based on the natural historical course of HBV infection (Additional file 1: Appendix p3), and the corresponding compartments or disease states are expanded according to the respective study objectives and characteristics. More details about these two models were exhibited in Additional file 1: Appendix p3–8.

### Interventions scenarios

During the course of the hepatitis B immunization program in China, the infant vaccination strategy, timely birth-dose vaccination strategy for newborns, and PMTCT strategies (e.g., the use of HBIG, antiviral treatment in childbearing-age patients with chronic HBV infection) have been successively implemented. Therefore, a real-world scenario (“Status quo”) was simulated for the combination of these strategies (e.g., 99.5% of the infant vaccination rate; 96.0% of timely birth-dose vaccination rate, etc.) in the above performed immunization program.

Furthermore, to assess the impact of the Chinese HBV immunization program strategy, we also modeled three additional immunization scenarios. One of the hypothetical scenarios we considered was a situation where the hepatitis B immunization program had never been conducted in China, which meant that all historical immunization interventions were removed (a scenario “Without any interventions”). An additional scenario simulated the introduction of the hepatitis B vaccine into the national immunization program and the waiver of vaccine costs, (84.3% of the infant vaccination rate; 66.1% of timely birth-dose vaccination rate), reflecting the impact of the program on the containment of hepatitis B in the early stages of implementation (a scenario “With the NIP”). The last scenario modeled the situation where hepatitis B vaccine (30.0% of infant vaccination rate; 22.2% of timely birth dose vaccination rate) had been rolled out in developing countries, yet the vaccine and services were paid for out-of-pocket as there was not included in the national immunization program and no financing (a scenario “Without the NIP”).

In addition, we further evaluated potential strategies that are feasible for the future. In turn, the magnitude of potential was assessed by the impact of each strategy on the prevalence of chronic HBV infections. Specifically, we estimated the impact of different levels of antiviral treatment of childbearing-age patients with chronic HBV infection (included in PMTCT strategies) on epidemic burden of HBV. In the modeling scenario, the range of antiviral treatment in childbearing-age patients with chronic HBV infection was set from 18 to 72%; while the impact of non-infant vaccination strategy was assessed in a range of 30–80% in non-infant vaccination rate. Both analyses were conducted in the status quo scenario of the year 2020 setting, which also included infant vaccination and timely birth-dose vaccination strategies.

### Economic analysis

The economic analysis of the relevant immunization strategies in the “Status quo” scenario was conducted through a decision tree-Markov model in TreeagePro2019 (TreeAge Software, Williamstown, MA). The decision tree part of the model was used to simulate the compliance and effectiveness of the infant vaccination strategy and PMTCT strategy of hepatitis B. The practice of scenario "Without any interventions" was set as the comparison group. The Markov part of the model was used to simulate HBV infection and progression (Additional file 1: Figs. S2, S3). The cycle length of each state was set to one year, and the termination condition of the cycle was the average life expectancy of Chinese in that year.

The parameters used in the model involved three parts, including costs, utilities and a series of model-related rates, which were mainly estimated based on published literature, government documents, and surveys conducted in this study. The cost parameters mainly include costs of hepatitis B vaccination and costs related to HBV infection. Hepatitis B vaccination costs include the cost of vaccine procurement, transportation and storage, which was based on our survey of CDC nationwide in 2018. HBV infection costs was from the survey of economic burden of hepatitis B related diseases in twelve cities in China [19], including outpatient expenses, hospital bed costs and the loss of income from missed work by the patient and accompanying persons. Since the survey was conducted in 2010, it was adjusted for inflation to 2018. The transition probabilities of different infection states after HBV infection and their utility values were mainly derived from literatures on China or the Western Pacific Region. Effectiveness was quantified by quality-adjusted life-years (QALYs), with the number of QALYs calculated by the product between life years and utilities by the TreeagePro2019. More detailed epidemiological parameters (the incidence of HBV infection in general population, the Infant vaccination rate, the HBIG coverage, etc.) were shown in the Additional file 1: Table S2.

The expected cost and effectiveness were obtained for each of the two strategies. A cost–benefit analysis (CBA) and cost-effectiveness analysis (CEA) were implemented by calculating indicators such as benefit–cost ratios (BCR) and incremental cost-effectiveness ratios (ICER).

### Sensitivity analyses

The sensitivity analysis of the dynamic model was completed by varying the main parameters, aiming to measure the impact of parameters on the number of chronic HBV infections and R_e_ (Additional file 1: Appendix p7). The sensitivity analysis of Markov model includes one-way sensitivity and probabilistic sensitivity analysis based on Monte-Carlo simulation with 10,000 iterations to evaluate the impact of parameter uncertainty on the ICER.

## Results

### Epidemic burden of hepatitis B in China

**Fig. 1:**
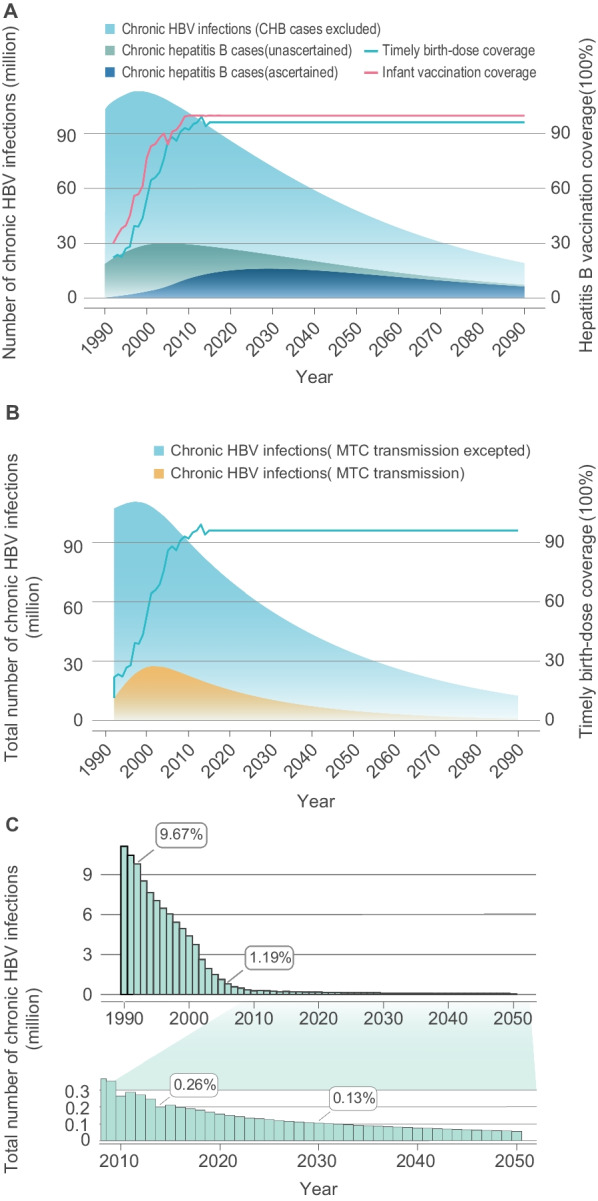
Estimated burden of chronic hepatitis B infection in China. **A** The number of chronic HBV infections and chronic hepatitis B (CHB) cases; **B** The estimated proportion of mother-to-child transmission account for the total number of chronic HBV infections; **C** the number of chronic HBV infections that were less than 5 years old. Note: chronic HBV infection is defined as a dynamic process with HBsAg and/or HBV DNA positivity for more than 6 months, reflecting the interaction between HBV replication and the host immune response; while CHB is defined as a chronic inflammatory disease of the liver caused by persistent HBV infection. *HBV* Hepatitis B virus

In tandem with increases in HBV vaccination rates, of which infant vaccination rate achieved 99.5% and timely birth-dose vaccination rate reached 96%, compared with 1992, the prevalence of total chronic HBV infections (CHB cases included) has declined by 33.9% in 2020 (70.83 million, nearly 5.02% prevalence). By 2020, there were 26.77 million CHB cases, of which there were 11.66 million unascertained and 15.10 million ascertained CHB cases, with the real-time ascertained rate of CHB cases increasing to 56%. Compared with 2020, the model predicted the prevalence of total chronic HBV infections will decrease to 55.73 million (3.95% prevalence) with a decline of 48.0% and the total number of CHB cases will decrease to 23.58 million with a decline of 12.0% in 2030 (Fig. 1A).

In addition, the maximum proportion that cases with chronic HBV infection caused by mother-to-child transmission account for the total number of chronic HBV infections is 25.6%. As predicted, it will decline to about 10.92 million by 2030, accounting for 20.0% of the total number of chronic HBV infections, which decreased by 31.1% compared with 2020 (Fig. 1B). As shown in Fig. 1C, the main beneficiaries of the infant vaccine immunization strategy are newborns and children: compared with 1990, the prevalence of total chronic HBV infections in those under 5 years old has decreased by 98.6% in 2020 (0.16 million, 0.20% prevalence).

### The feasibility of the WHO 2030 elimination goals

**Fig. 2:**
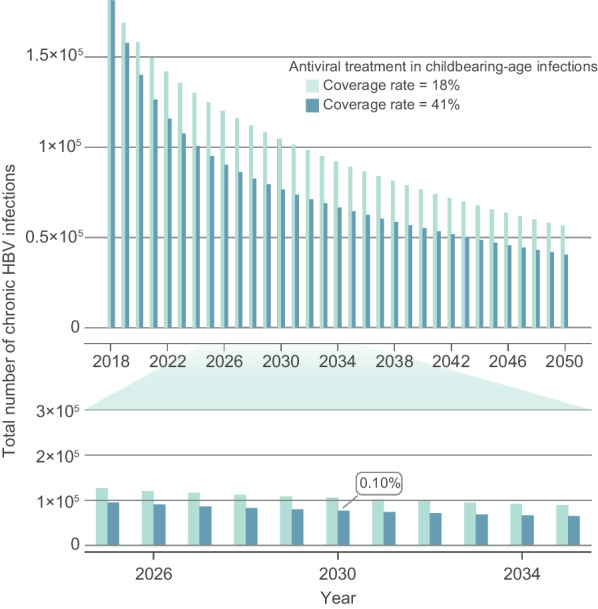
Exploration of strategies to achieve less than 0.1% prevalence among children under five by 2030. Estimation of the potential of expanding the coverage rate of antiviral treatment among childbearing-age patients with chronic HBV infection (including in PMTCT strategies) on achieving the 2030 Goals. *HBV* Hepatitis B virus, *PMTCT* Prevention of mother to child transmission

Due to the immunization strategies initiated by health facilities and the Chinese government, the coverage rate of HBV vaccine in newborns has been greatly increased; the infant coverage rate of HBV vaccine has increased from 30.0 to 99.5% from 1992 to 2018; the timely birth-dose coverage of HBV vaccine reached 96% in 2018 (Fig. 1A). Vaccination uptake has already achieved service coverage target of the WHO “2030 Elimination Goals”. Additionally, the model predicted that if high vaccination coverage was kept consistent after 2020, the prevalence of chronic HBV infections that were under 5 years old would decrease further to 0.13% by 2030 (Fig. 1C). Furthermore, we found the prevalence of chronic HBV infections could drop below 0.1% with the increase the coverage of antiviral treatment in childbearing-age patients with chronic HBV infection to 41% (Fig. 2). The findings of potential immunization strategies to fulfill WHO 2030 target of 90% incidence reduction exhibited in Additional file 1: Appendix p10.

### The impact of immunization strategies of hepatitis B

**Fig. 3:**
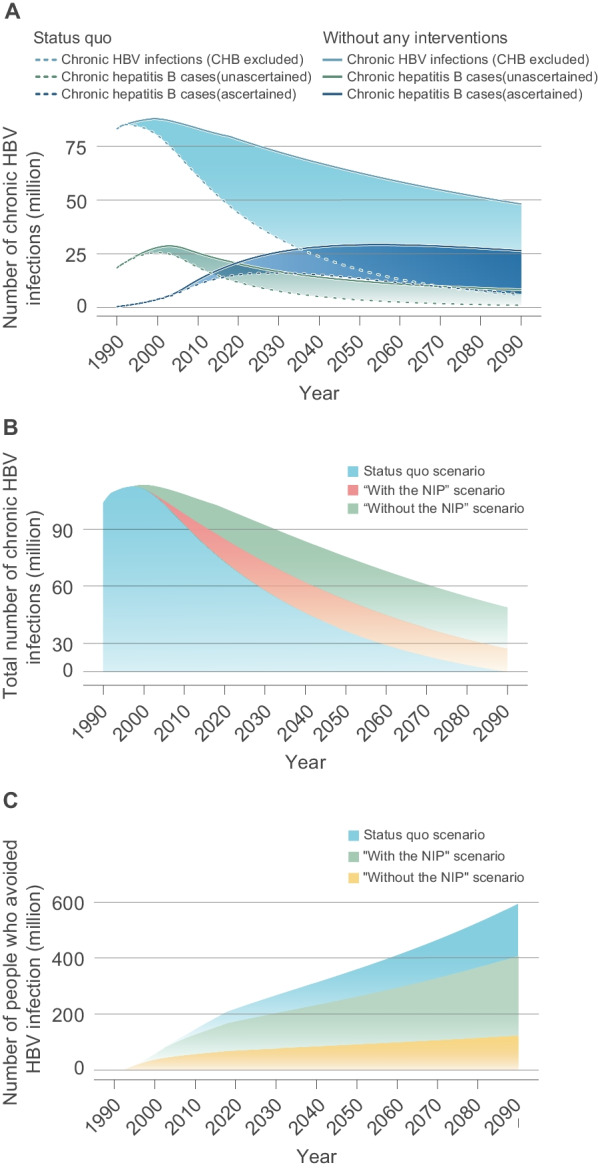
Effect of immunization interventions on the hepatitis B epidemic in China. **A** The prevalence of chronic HBV infections and chronic hepatitis B (CHB) cases in the “Status quo” scenario and “Without any interventions” scenario, respectively. **B** The total prevalence of chronic HBV infections which included CHB in the “Status quo” scenario, “With the NIP” scenario and a scenario “Without the NIP”. **C** The number of people who avoided HBV infection. More information about the definitions of these four scenarios exhibited in “Method” section. *HBV* Hepatitis B virus

Model results were compared across the four scenarios (see Methods). In the “Status quo” scenario, there is a number of 44.07 million chronic HBV infections (CHB cases excluded) estimated in 2020. The results showed removing all the interventions (“Without any interventions” scenario) would have led to 78.21 million chronic HBV infections (CHB cases excluded) and nearly 77.5% increase compared with Status quo by 2020. Keeping interventions constant with the scenario “Without the NIP” and the scenario “With the NIP” would lead to 99.40 million and 82.67 million chronic HBV infections, with increase of 40.3% and 16.7% compared with Status quo by 2020, respectively. The total number of CHB cases in the “Without any interventions” scenario, the scenario “Without the NIP” scenario and the scenario “With the NIP” would have increased to 41.32 million, 35.19 million and 30.17 million in 2020, respectively; however, the total number of CHB cases was 26.27 million in status quo scenario, shown in Fig. 3A, B. Based on the model at the status quo scenario, it avoided 217.6 million individuals to infect HBV due to hepatitis B immunization strategies in the past 30 years, while in the scenarios maintained with the scenario “Without the NIP” and the scenario “With the NIP”(lower HBV vaccination coverage rates than Status quo scenario), it only avoided 70 million and 172.6 million individuals with HBV infection, respectively (Fig. 3C).

**Fig. 4:**
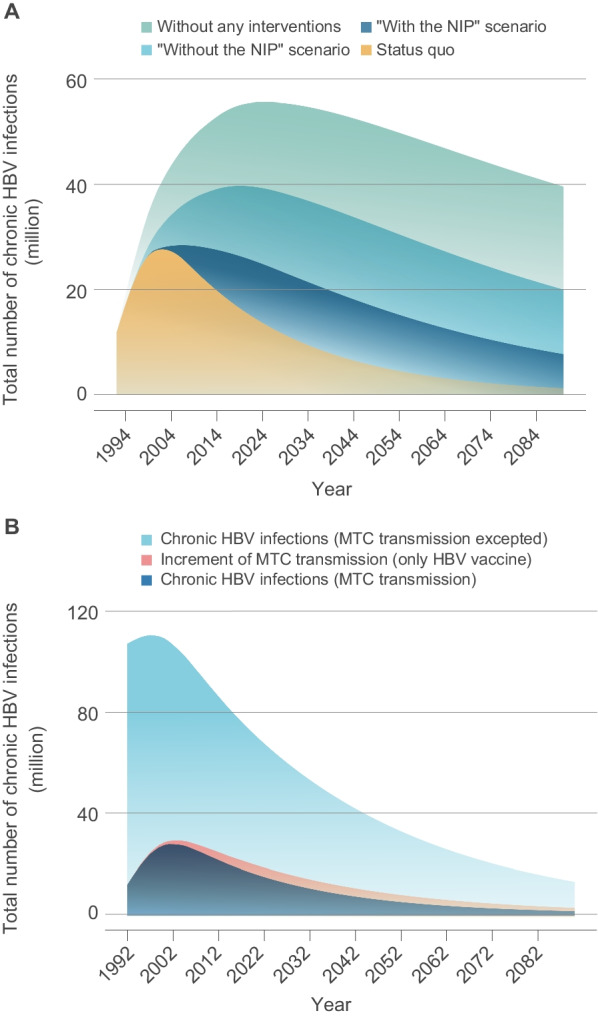
Effect of immunization interventions of hepatitis B on mother-to-child transmission. **A** The total prevalence of chronic HBV infection through mother-to-child (MTC) transmission in the “Status quo” scenario, “With the NIP” scenario, “Without the NIP” scenario and a scenario “Without any interventions”, respectively. **B** The total prevalence of chronic HBV infections (Light blue area) and chronic HBV infection tMTC transmission (Blue area) in the “Status quo” scenario, respectively; the increment of chronic HBV infections by MTC transmission was exhibited in the “pink area”. *HBV* Hepatitis B virus, *NIP* National Immunization Program

Chronic HBV infections resulting from mother-to-child transmission declined substantially in response to interventions over the past 30 years. The number of chronic HBV infections infected by the mother-to-child transmission route decreased from a projected 55.43 million in the scenario with all immunization strategies removed, to 39.82 million when immunization strategies maintained the scenario “Without the NIP”, to 26.18 million when immunization strategies maintained with the scenario “Without the NIP”, to 15.84 million in the status quo in 2020 (Fig. 4A). Additionally, it would add 3.69 million chronic HBV infections in 2020, if the antiviral treatments and immunoglobulin interventions were removed from the PMTCT strategy (Fig. 4B).

### Economic projections

The model was run with a cycle length of one year to the average life expectancy of Chinese (i.e., from birth to 77 years old) in order to cover lifelong experiences of the vast majority of the newborns. The cost–benefit analysis in Table 1 showed that the total cost per capita with the current infant vaccination strategy is United Sates Dollar (USD) 1260.78 in “Status quo” scenario, whereas the “Without any interventions” scenario had a cost per capita of USD 13,177.03. The implementation of the infant vaccination strategy added USD 52.17 in immunization costs, but reduced costs related to HBV infection by USD 11,968.42, for a net benefit of USD 11,916.25. In “Status quo” scenario, the PMTCT strategy showed net benefit as USD 11,283 per person, with BCR as 12.66, which was higher than that of infant vaccination strategy at 9.49. On the basis of 10.03 million newborns born in China in 2020, a total of 0.52 billion was needed to implement the infant vaccination strategy, generating benefits of approximately 120.10 billion.

**Table 1:** Cost benefit and cost-effectiveness analysis of infant hepatitis B vaccine and PMTCT, compared to no intervention

Strategy	Cost (USD)			QALYs	Benefit	NB	BCR
	Immunization	Infection	Total				
Status quo							
Infant hepatitis B vaccine	52.17	1208.61	1260.78	23.88	11,968.42	11,916.25	9.49
PMTCT	76.37	893.52	969.89	23.92	12,283.50	12,207.14	12.66
Comparison scenario							
Without any interventions	0	13,177.03	13,177.03	22.34	–	–	–

Note: All costs were adjusted with respect to the cost of dollar according to the exchange rate in 2018

*USD* US dollar, *QALY* quality-adjusted life year, *NB* net benefit, *BCR* benefit–cost ratio, *PMTCT* the prevention of mother-to-children transmission

The cost-effectiveness analysis showed that the implementation of the infant vaccination strategy and PMTCT strategy can increase 1.54 and 1.58 QALYs per person, respectively, in the “Status quo” scenario. Compared with the scenario “Without any interventions”, the former could save $7737.82 for each QALY increase, and the latter could save $7726.03, indicating they are both cost-effective intervention measures. Compared with the infant vaccination strategy, the cost of the PMTCT decreased by $290.89, and the effect increased by 0.04 QALY, which further reduced costs while reducing infections among infants.

### Sensitivity analysis

**Fig. 5:**
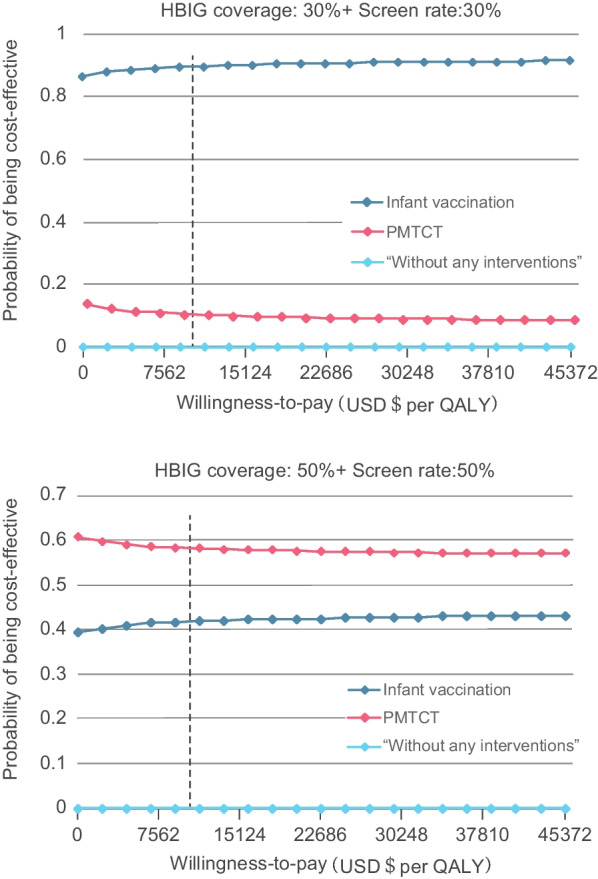
Cost-effectiveness acceptability curves for immunization strategies. Cost-effectiveness acceptability curves of the infant vaccination strategy (blue curve) and the PMTCT strategy (red curve) under 50% and 30% HBIG coverage and screening rate. *USD* US dollar,  *HBIG* hepatitis B immunization program, *QALY* quality-adjusted life year, *PMTCT* the prevention of mother-to-children transmission

According to the one-way sensitivity analysis with the range of values shown in Additional file 1: Table S2, both of the infant vaccination strategy and PMTCT strategy strategies were cost-saving. Even if the vaccination rate was adjusted to 0.3, as it was in 1992, the infant vaccination strategy still reduced costs related to HBV infection by $3794.98. The probabilistic sensitivity analysis in Additional file 1: Fig. S8 showed that when the parameters varied within the set value range, the ICER of the PMTCT strategy compared with the infant vaccination strategy was always cost-saving, and the results were very stable. We continued to observe the economic benefits of the two strategies by continuously reducing the prenatal screening rate and the HBIG coverage rate. Figure 5 showed that when the two parameters were down to 30%, compared with PMTCT, the infant vaccination strategy was more cost-effective even at a WTP (Willingness-to-pay) of one-times the per-capita GDP.

## Discussion

During the past three decades, given that China implemented comprehensive immunization strategies to contain the transmission of HBV, we used mathematical models to evaluate the health and economic impact of immunization strategy carried out in China. The major results of model estimation and prediction showed that the immunization strategies exhibited dramatic economic and health benefits avoiding a large number of HBV infections, HBV-related diseases and death, and improving the quality of life. Even with continuous efforts applied to the prevention and control, the goal of a 90% reduction in incidence of new chronic infections would be missed by a small gap (0.03%). To fulfill this target, we explore potential directions and found it could be achieved with enhancement the PMTCT strategy. The CEA and CBA results also showed that the strategies of infant vaccination and PMTCT in status quo can achieve more economic and health benefits than “Without any intervention” scenario.

From the results of CBA, although the BCR of PMTCT was higher than that of infant vaccination strategy, it was also found that the cost savings for each additional QALY of the infant vaccination strategy was higher than the cost saved by the PMTCT, indicating that universal vaccination was indispensable stage for PMTCT strategy. Economic evaluations from Italy [20], Vietnam [21], Iran [22] have shown that the infant vaccination on HBV is a cost-effective strategy. Combined with the results of the dynamic model, the practical effect of universal vaccination in China also suggests that PMTCT strategy not only emphasizes the importance of active and passive immunization for newborns coming from pregnant women infected with HBV, but emphasizes the importance of early standardized HBV vaccination for all newborns. For countries with limited human resources and financial support, especially some high disease burden regions in which proportion of childbearing-age women delivered by health staff was only 50% or less, such as sub-Saharan Africa [23] and South-East Asia [24], priority should be given to the implementation of the infant HBV vaccination strategy [25, 26].

The model estimated that the number of cases with chronic hepatitis B infection and HBsAg positive rate in China have been substantially reduced to 78.83 million and 5.33% by 2020, respectively. These results are consistent with another global study [27]. However, the decline in CHB was only a third as large as the reduction in the number of chronic HBV infections. The underlying reason could be that universal HBV vaccination strategy against hepatitis B is relatively late and the diagnosis rate and treatment rate are relatively low [9, 28], which is common in the low- and middle-income countries most affected by HBV endemicity. Due to the complexity and cost for the diagnosis and management of hepatitis B, millions of individuals are unaware of their infection, of which in need of treatment only extremely low proportion is receiving antiviral therapy [11, 27]. Also, some research pointed out the education of HBV knowledge and awareness were still limited in general population [29] so far which could cause delay of testing and treatment [30].

Although the prevalence of chronic HBV infection in China has been effectively controlled, it would keep a median high prevalence level by projection at least for 10 years. Moreover, mother-to-child transmission remains a high-risk route [31] and it’s associated with an increased risk of liver complications which would lead to more deaths [32, 33]. Therefore, the PMTCT work should be prioritized and strengthened in the further containment of HBV in the future. In details of actions, expanding the coverage of screening and reinforcing antiviral treatment for infected women of childbearing age are required for HBV elimination.

The strength of our study is that we constructed a dynamic model, which took the under-reported situation of surveillance data in the 1990s into consideration. Such consideration enables the model to more realistically estimate the historical burden of hepatitis B; and strengthens the robustness of the predicted results for the changes in future epidemics. Another strength is that we evaluated the impact of different HBV immunization strategies and the feasibility of achieving the WHO 2030 elimination goal by comparing real-world scenario with multiple counterfactual immunization scenario. The evidence from this study not only facilitates the optimization of containment strategies in China, but also extends the Chinese containment experience of hepatitis B to similar low- and middle-income countries with a high burden of chronic HBV infection, especially those regions that still have relatively low vaccine coverage due to unequal vaccine distribution and poor equity. However, there is a limitation that we couldn't include this cost of safe administration expenditure in the analysis of our study, due to the data on administration expenditure wasn't available in open databases.

## Conclusions

The infant vaccination and PMTCT strategies implemented in China's hepatitis B immunization program have played an essential role in the HBV containment, which have effectively controlled the prevalence of chronic HBV infections. Both of the strategies were cost-effective, improving quality of life and reducing costs of care. The upper limited effectiveness of vaccine-related strategies and the remaining gap in the "2030 Elimination Goals" both indicated that it's essential and urgent to further explore potential containment strategies in addition to maintaining the current level of immunization strategies. Based on the projection of our study, the achievement of the global “2030 Elimination Goals” could be considerably accelerated under the further enhancement of a PMTCT strategy.

## Supplementary Information

**Additional file 1: Fig: S1.** Transmission structure of the HBV mathematical model.** Fig. S2. **HBV infection and progression structure of Markov model.** Fig. S3. **Process of acute HBV infection. **Fig. S4. **Dynamic model fitting and prediction results. The blue points represent the observed incidence data, while the black cross represents the incidence data fitted by the model. Red triangles represent the predicted incidence data. **Fig. S5. **Exploration of feasible vaccination interventions on HBV. A: the impact of varied coverage rates of antiviral treatment in childbearing-age patients with chronic HBV infection and non-infant vaccination coverage on the total chronic HBV infections; B: the impact of varied coverage rates of antiviral treatment in childbearing-age patients with chronic HBV infection and non-infant vaccination coverage on the MTCT cases; C: the impact of varied coverage rates of antiviral treatment in childbearing-age patients with chronic HBV infection and non-infant vaccination coverage on the chronic HBV infections under five years old; D: the impact of varied coverage rates of antiviral treatment in childbearing-age patients with chronic HBV infection and non-infant vaccination rate on the number of averted infections. MTCT: mother-to-child transmission. **Fig. S6. **Sensitivity analysis of parameters and R_e_. Estimated impacts of parameters on the R_e_, with varied ranges based on sensitivity analysis. A, B, C, D show the parameters (β_2_, β_3_, D_c_, e_2_, θ, ω_2_) range below to above of their setting values, respectively. **Fig. S7. **Sensitivity analysis results of parameters. Estimated impacts of parameters on the number of chronic HBV infections (I_c_ + I_u_ + I_d_), with varied ranges based on sensitivity analysis. A, B, C, D show the four parameters (β_2_, β_3_, D_c_, e_2_) range below to above of their setting values, respectively. **Fig. S8. **Results of Probabilistic Sensitivity Analysis. Incremental Cost-Effectiveness among the PMTCT strategy vs. Infant vaccination strategy (conducted in “Status quo” scenario). **Table S1. **Critical parameters of HBV mathematical model.** Table S2. **Parameters of decision tree-Markov model. **Table S3. **Time-varied data of birth, death and vaccination rates.

## Data Availability

The datasets generated and analyzed during the current study are available in the https://github.com/wwbgroup/Hepatitis-B_national-immunization-program.

## Abbreviations

HBV: Hepatitis B virus
HBsAg: Hepatitis B surface antigen
CHB: Chronic hepatitis B
NIP: National Immunization Program
WHO: World Health Organization
CDC: Center for Disease Control and Prevention
NNDRS: National Notifiable Disease Reporting System
UNICEF: United Nations International Children's Emergency Fund
PMTCT: Prevention of mother to child transmission
SEIR: Susceptible-exposed-infectious-recovered
ICER: Incremental cost effectiveness ratio
BCR: Benefit–cost ratio
CBA: Cost benefit analysis
CEA: Cost effectiveness analysis
WTP: Willingness-to-pay
QALYs: Quality-adjusted life-years
