# Supplementary material for: Impact of the national hepatitis B immunization program in China: a modeling study
Source: Infect Dis Poverty. 2022 Oct 11;11:106. doi: 10.1186/s40249-022-01032-5 (PMC9552421; doi:10.1186/s40249-022-01032-5)
Supplement: Supplementary file 1 — Additional file 1: Fig: S1. Transmission structure of the HBV mathematical model. Fig. S2. HBV infection and progression structure of Markov model. Fig. S3. Process of acute HBV infection. Fig. S4. Dynamic model fitting and prediction results. The blue points represent the observed incidence data, while the black cross represents the incidence data fitted by the model. Red triangles represent the predicted incidence data. Fig. S5. Exploration of feasible vaccination interventions on HBV. A: the impact of varied coverage rates of antiviral treatment in childbearing-age patients with chronic HBV infection and non-infant vaccination coverage on the total chronic HBV infections; B: the impact of varied coverage rates of antiviral treatment in childbearing-age patients with chronic HBV infection and non-infant vaccination coverage on the MTCT cases; C: the impact of varied coverage rates of antiviral treatment in childbearing-age patients with chronic HBV infection and non-infant vaccination coverage on the chronic HBV infections under five years old; D: the impact of varied coverage rates of antiviral treatment in childbearing-age patients with chronic HBV infection and non-infant vaccination rate on the number of averted infections. MTCT: mother-to-child transmission. Fig. S6. Sensitivity analysis of parameters and Re. Estimated impacts of parameters on the Re, with varied ranges based on sensitivity analysis. A, B, C, D show the parameters (β2, β3, Dc, e2, θ, ω2) range below to above of their setting values, respectively. Fig. S7. Sensitivity analysis results of parameters. Estimated impacts of parameters on the number of chronic HBV infections (Ic + Iu + Id), with varied ranges based on sensitivity analysis. A, B, C, D show the four parameters (β2, β3, Dc, e2) range below to above of their setting values, respectively. Fig. S8. Results of Probabilistic Sensitivity Analysis. Incremental Cost-Effectiveness among the PMTCT strategy vs. Infant vaccination strategy (condu [file 40249_2022_1032_MOESM1_ESM.docx]

**Impact of the national Hepatitis B immunization program in China: a modeling study**

**Appendix**

**Contents**

1. Terminology Definitions
   1. Chronic hepatitis B virus infection (Chronic HBV infection)
   2. Chronic hepatitis B (CHB)
2. Model Description

2.1 Model Conceptions

- - 1. Natural History of HBV Infection
  1. Mathematical Model
     1. Mathematical Model Framework
     2. Parameter Settings and Initial States
     3. Differential Equations
     4. Parameter Estimation and Model Fitting
     5. Derivation and Calculation of Reproductive Number (R_e_)
     6. Sensitivity Analysis
  2. Decision Tree-Markov Model
     1. Decision Tree-Markov Model Framework
     2. Force of Infection

1. Results of Mathematical Model
   1. Mathematical Model Fits
   2. Calculation of Reproductive Number (R_e_)
   3. Potential immunization strategies
   4. Sensitivity Analysis

**SUPPLEMENTARY TABLES**

**Table S1: Critical parameters of HBV mathematical model**

**Table S2:** **Parameters of decision tree-Markov model**

**Table S3:** **Time-varied data of birth, death and vaccination rates**

**SUPPLEMENTARY FIGURES**

**Fig S1: Transmission structure of the HBV mathematical model.**

**Fig S2: HBV infection and progression structure of Markov model**

**Fig S3: Process of acute HBV infection**

**Fig S4: Dynamic model fitting and prediction results.** The blue points represent the observed incidence data, while the black cross represents the incidence data fitted by the model. Red triangles represent the predicted incidence data.

**Fig S5: Exploration of feasible vaccination interventions on hepatitis B.** A: the impact of varied coverage rates of antiviral treatment in childbearing-age patients with chronic HBV infection and non-infant vaccination rates on the total chronic HBV infections; B: the impact of varied coverage rates of antiviral treatment in childbearing-age patients with chronic HBV infection and non-infant vaccination rate on the MTCT cases; C: the impact of varied coverage rates of antiviral treatment in childbearing-age patients with chronic HBV infection and non-infant vaccination rates on the chronic HBV infections under five years old; D: the impact of varied coverage rates of antiviral treatment in childbearing-age patients with chronic HBV infection and non-infant vaccination rates on the number of people who avoided HBV infection. MTCT: mother-to-child transmission.

**Fig S6: Sensitivity analysis of parameters and R_e_.** Estimated impacts of parameters on the R_e_, with varied ranges based on sensitivity analysis. A, B, C, D show the parameters (β_2_, β_3_, D_c_, e_2_, θ, ω_2_) range below to above of their setting values, respectively.

**Fig S7: Sensitivity analysis results of parameters.** Estimated impacts of parameters on the number of chronic HBV infections (I_c_+I_u_+I_d_), with varied ranges based on sensitivity analysis. A, B, C, D show the four parameters (β_2_, β_3_, D_c_, e_2_) range below to above of their setting values, respectively.

**Fig S8: Results of Probabilistic Sensitivity Analysis**

Incremental Cost-Effectiveness among the PMTCT strategy vs. Infant vaccination strategy (conducted in “Status quo” scenario).

1. **Terminology Definitions**

**1.1 Chronic hepatitis B virus infection (Chronic HBV infection)** is defined as a dynamic process with HBsAg and/or HBV DNA positivity for more than 6 months, reflecting the interaction between HBV replication and the host immune response.

**1.2 Chronic hepatitis B (CHB)** is defined as a chronic inflammatory disease of the liver caused by persistent HBV infection.

Noted, not all patients with chronic HBV infection can be defined as chronic hepatitis B (CHB), only the portion of chronic HBV infections who were accompanied with chronic inflammatory liver disease can be classified to this category. Thus, CHB cases are belong to a part of chronic HBV infections; and the total number of chronic HBV infections is defined as the sum of chronic HBV infections without chronic inflammatory liver disease and CHB cases.

1. **Model Description**

As described in the main text, after completing the estimation of the epidemic burden of chronic HBV infections in China, this study further evaluated the achievement of the 2030 goal by predicting the real-time total number of chronic HBV infections and then completed the impact evaluation of immunization strategies. Finally, cost-benefit and cost-effectiveness analysis were used to evaluate the effectiveness of immunization strategies from the perspective of economics.

**2.1 Mathematical Conceptions**

**2.1.1 Natural History of HBV Infection**

The model framework is based on this natural historical course of HBV infection: once HBV infection is established in a host, the clinical illness may take one of two possible courses[1]: acute infection or chronic infection. The average duration of an acute HBV infection is usually 3 months. Therefore, if an acute HBV infectious individual doesn’t self-clear HBV or if the duration exceeds 6 months, they will enter the chronic infection stage. Not all chronic HBV infection can be diagnosed as chronic hepatitis B (CHB). Chronic HBV infection can be defined as being HBsAg and/or HBV DNA positive for at least 6 months, while CHB is a chronic inflammatory disease of the liver caused by persistent HBV infection[2]. In a person with chronic HBV infection, the liver is constantly exposed to virus, and the condition may progress to liver cirrhosis. If cirrhosis has not been treated in time, patients may develop complications of cirrhosis such as edema, jaundice and ascites (called decompensation). A few individuals with chronic HBV infection individual can gain immunity shifting to an immune state.

**2.2 Mathematical Model**

**2.2.1 Mathematical Model Framework**

In this study, the whole population was divided into nine compartments to represent each stage of hepatitis B based on the characteristics of transmission, natural history and literature. In addition, due to omitted report of HBV infections and lack of standardized diagnostic criteria in the 1990s, the number of reported infections is substantially smaller than the realistic infections. Therefore, it’s crucial to divided CHB compartment into ascertained CHB (the CHB cases were caught and reported by the National Notifiable Disease Report System (NNDRS) and unascertained CHB (the CHB cases that were omitted and failed to report by the NNDRS) compartment for interpreting transmission dynamics and epidemic of HBV infections. Specifically, we described the dynamic progress of hepatitis B by extending the susceptible-exposed-infectious-recovered (SEIR) model to include age-varied acute infectious (A_i_) (Given to differences in the risk of progression to chronic disease among acute infectious individuals at different age ranges), chronic HBV infection (I^c^), unascertained CHB (I^u^) and ascertained CHB (I^d^), generating a mathematical model to estimate the burden and epidemic trend of hepatitis B during the past 30 years. The model framework is shown in **Fig S1**.

**2.2.2 Parameter Settings and Initial States**

We obtained critical parameters for our model through research literature and government reports. Parameter settings for the analysis are summarized in S1 Table. Based on the sero-epidemiological survey

of hepatitis B conducted in 1992, the total number of chronic HBV infection Ic(0), unascertained CHB Iu(0) and ascertained CHB Id(0) were estimated as the prevalence data on hepatitis B surface antigen (HBsAg) multiplied by the total population size, the number of recovery cases R(0) was estimated as the prevalence data of hepatitis B core antibody (Anti-HBc) multiplied by the total population size. Further, the initial acute HBV infections A<1 years old (0), A1-5 years old (0) and A>5 years old (0) was estimated by use corresponding data from the institute for health metrics and evaluation (IHME). The detailed model is described by a set of differential equations showed in Appendix p5. Furthermore, the parameter “θ” was set to assume coverage rate of antiviral treatment among childbearing-age patients with chronic HBV infection (as a part of PMTCT strategies); the baseline of chronic HBV infection in women of gestational age in the general population (parameter b) was quantified by calculating the proportion of women of gestational age with chronic HBV infection in the general population of women in the same age group; then we obtained *P_A_* = (1-θ)×b, which reflect the impact of antiviral treatment among childbearing-age patients with chronic HBV infection on the containment of mother-to-child transmission. Therefore, this parameter will decrease with the expansion of standard treatment for women of childbearing age and vice versa.

**2.2.3 Differential equations**

$$\frac{dS}{dt}=\mu\omega_{1}\left[ N-P_{A}\left( I^{c}+I^{u} \right) \right]+\mu{(1-e}_{1})\left( 1-\omega_{1} \right)\left[ N-\theta\left( I^{c}+I^{u} \right) \right]-\frac{\left[ \beta_{1}\left( A_{1}+A_{2}+A_{3} \right)+{\beta_{2}I}^{c}+\beta_{3}I^{u} \right]S}{N}-e_{3}p(t)S+\varphi S-kS$$

$$\frac{dE}{dt}=\frac{\left[ \beta_{1}\left( A_{1}+A_{2}+A_{3} \right)+{\beta_{2}I}^{c}+\beta_{3}I^{u} \right]S}{N}-D_{e}E$$

$$\frac{dA_{<1 years old}}{dt}=\rho_{1}D_{e}E-{D_{a}A}_{1}$$

$$\frac{dA_{1-5 years old}}{dt}=\rho_{2}D_{e}E-{D_{a}A}_{2}$$

$$\frac{dA_{>5 years old}}{dt}=\left( 1-\rho_{1}-\rho_{2} \right)D_{e}E-{D_{a}A}_{3}$$

$$\frac{dI^{c}}{dt}=q_{i}D_{a}A_{i}+\mu\omega_{2}P_{A}I^{c}+\mu\left( {1-e}_{2} \right)\left( 1-\omega_{2} \right)P_{A}I^{c}+\gamma_{1}\left( I^{u}+I^{d} \right)-{D_{c}I}^{c}-kI^{c}-\gamma_{c}I^{c},(i=1,2,3)$$

$$\frac{dI^{u}}{dt}=\left( 1-r_{\left( t \right)} \right)D_{c}I^{c}+\mu\omega_{2}P_{A}I^{u}+\mu\left( {1-e}_{2} \right)\left( 1-\omega_{2} \right)P_{A}I^{u}-{\gamma_{1}I^{u}-\gamma}_{2}I^{u}-kI^{u}-\gamma_{d}I^{u}-\gamma_{u}I^{u}$$

$$\frac{dI^{d}}{dt}=r_{\left( t \right)}D_{c}I^{c}-\gamma_{1}I^{d}-\gamma_{2}I^{d}-kI^{d}$$

$$\frac{dR}{dt}=\mu e_{1}\left( 1-\omega_{1} \right)\left[ N-P_{A}\left( I^{c}+I^{u} \right) \right]+\mu e_{2}\left( 1-\omega_{2} \right)P_{A}\left( I^{c}+I^{u} \right)+e_{3}p(t)S+\left( 1-q_{i} \right)D_{a}A_{i}+\gamma_{2}(I^{d}+I^{u})-\varphi S-kR$$

**2.2.4 Parameter Estimation and Model Fitting**

The yearly reported CHB data (a portion of the total number of HBV cases) from 1990 to 2018 are used to estimate β_1_, β_2_, and β_3_, among which represent the transmission rates for age varied acute infectious individuals, chronic infectious individuals, unascertained and ascertained CHB individuals, respectively. These parameters were estimated by minimizing the sum of squares (MSS); the whole fitting process was completed within the Fminsearch function package of the MATLAB R2019a tool, which is used to perform unconstrained nonlinear minimization to obtain all optimal parameters when the results of Fminsearch converged.

$MSS=sqrt(\sum\left[ I^{d}(i)-x(i) \right]$^2^

Where I^d^(i) indicates the fitted CHB data in year i; x(i) indicates the observed CHB data in year i. In addition, i represents the year i corresponding to the observed data of CHB. Furthermore, in order to test the goodness of fit between the estimated incidence data and the reported incidence data, a two-sample *t*-test was performed on the two groups of data. Null hypothesis (H_0_): The modeled incidence data (I^d^) is equal to the observed incidence data. Alternative hypothesis (H_1_): The modeled incidence data (I^d^) is not equal to the observed incidence data.

**2.2.5 Derivation and Calculation of Reproductive Number (R_e_)**

Effective Reproductive number (R_e_) is defined as the number of second-generation infectious cases caused by an infected individual in a susceptible population. It’s also a threshold indicator to measure the ability of HBV to invade a population and to determine the disease-free equilibrium (DFE). Based on the differential equations of the Hepatitis B dynamics model, the matrix expression of R_e_ was derived. If R_e_ < 1, it means that an infected individual has produced less than one new infection during his or her infection period, and the hepatitis B epidemic cannot increase. On the contrary, if R_e_ >1, each infected individual will produce more than one newly infected individual on average, and the disease will continue to exist and develop into a endemic epidemic or even an outbreak. Therefore, the calculation of this index plays an important role in judging the effect of the immunization strategy on the control of hepatitis B epidemic.

Considering that the dynamics model of hepatitis B is not a simple linear chain model in this study, the Next Generation Matrix method is adopted. The method was established in 1990 by Diekmann et al.[3], and it’s a general method for calculating reproductive number in any complex compartment model[4]. The method consists of the following steps:

(1) Based on the differential equations of hepatitis B dynamics model, identify all infectious compartments M;

(2) An M×1 matrix *f* was constructed based on the differential equations of all infectious compartments, including the expression of new infections caused by each infectious compartment and entering it. This matrix represents the occurrence rate of new infections in susceptible population.

(3) An M×1 matrix *ν*^+^ was constructed based on the differential equations of all infectious compartments, including the incremental expression of non-newly infectious cases entering each infectious compartment;

(4) An M×1 matrix *ν*^-^ was constructed based on the differential equations of all infectious compartments, including the expression of the loss amount of non-newly infectious cases entering each infectious compartment;

(5) By combining steps (3) and (4), an M×1 matrix ν was constructed, *ν* = *ν*^-^- ν^+^, which represented the progression rates of non-newly infected quantities in the corresponding infectious compartments.

(6) Calculate the Jacobian matrix for the matrix *f* and *ν* in step (2) and step (5) respectively, and get two m×m new matrix F and V, which represent the partial derivatives of matrix *f* and matrix *ν* with respect to m infectious state variables respectively;

(7) To obtain the inverse matrix V^-1^ of the matrix V;

(8) Solve the maximum eigenvalue of the spectral radius of the product of matrix F and V^-1^ to get the value of R_e_.

The calculation process was completed by Wolfram Mathematica (version 12.0) and MATLAB software (version R2019a).

**2.2.6 Sensitivity Analysis**

Sensitivity analysis was performed by changing the main parameters. According to the formula of basic reproductive number, we found out the relevant parameters that will affect R_e_. In order to further explore the relationship between R_e_ and these parameters, we conducted sensitivity analysis to measured and quantified the impact of these parameters (p, θ, β_2_, β_3_, D_c_ and e_2_) on the number of chronic HBV infections and R_e_. In the analysis, the transmission rate of chronic HBV infections (β_2_) is set as 0.031-0.60; the transmission rate of CHB cases (β_3_) of 0.41-0.021. The rate of chronic HBV infections becoming CHB (D_c_) was 0.1-0.0025. The efficacy of timely birth-dose HBV vaccine (e_2_) on mother-to-child transmission of 70–99% (the second value is the default value used in the model). The vaccination coverage of non-newborn (p) varied from 30-80% since 2018 (the first value is the default value used in the model). The theta (θ) value varied from 18%-72% since 2018 (the second value is the default value used in the model. The ranges of these parameters were set to quantify the capacity of immunization strategies that could be improved in the future. All the above analyses were performed by MATLAB software (version number: R2019a).

**2.3 Decision Tree-Markov Model**

**2.3.1 Decision Tree-Markov Model Framework**

The Markov part of the model was used to simulate HBV infection and progression. It was mainly constructed based on "China's Diagnostic criteria for hepatitis B virus (ws299-2008)", "Diagnostic criteria for chronic hepatitis B (2015)" and relevant literature at home and abroad. This section includes a total of eight Markov states: susceptible to HBV; recovery due to infection or vaccination; chronic HBV infection; chronic hepatitis (CHB); compensated cirrhosis; decompensated cirrhosis; hepatocellular carcinoma; and death. The relationships between them are shown in **Fig S2**. In addition, "acute infection" was not a Markov state in this model, which was a short process in the " susceptible to HBV " state. The process of acute HBV infection was shown in **Fig S3**.

**2.3.2 Force of Infection**

Formula 1:$P\left( a \right)=k(1-e^{(\frac{b_{2}}{b_{3}}\left( 1-e^{b_{3}a}-b_{1}a \right))}$

Formula 2:$\lambda\left( a \right)=b_{1}+b_{2}\times e^{\left（ b_{3}a \right）} (b_{1}>0,b_{2}>0,b_{3}<0)$

Formula 3:$\lambda\left( a \right)=0.034435+0.49448\times e^{（-0.729443\times age）}$

Because the incomplete data on the incidence of acute hepatitis B reported in China, this study estimated the annual incidence of HBV infection in susceptible populations of different age groups based on the data from the national sero-epidemiological. As the sero-epidemiological survey in 1992 was performed just before the recommendation of routine hepatitis B vaccination for newborns was announced, its data could characterize natural HBV infections without vaccination. The catalytic model was used to fit the national data, and the best fitting was the age-dependent exponential equation as shown above. Formula 1 and 2 are the original catalytic models used to fit the national data. Formula 3 is the fitting result. *P(a)* is the prevalence of HBV infection in the population at age a. A person with a positive test result for HBV surface antigen (HBsAg), core antibody (anti-HBc) or surface antibody (anti-HBs) was defined as one who had been infected by HBV. λ(a), as a function of age, denotes the age-dependent force of HBV infection.

**3. Results of Mathematical Model**

**3.1 Mathematical Model Fits**

The fitting result of estimated incidence data through modeling is basically consistent with the observed data reported by the NNDRS, as shown in **Fig S4**. The result of two-sample *t*-test shows the *p* value is 0.985 which is greater than 0.05; therefore, we can’t reject the null hypothesis (H_0_) at the 5% significant level. The modeled result is a good estimate of the observed data.

**3.2 Calculation of Reproductive Number (R_e_)**

1. It can be seen from the model equations that there are 6 infectious compartments in this model, which are E, A_<1 years old_, A_1-5 years old_, A_>5 years old_, I^c^ and I^u^ respectively. Thus, two 6×1 matrices *f*, *ν*^+^ and *ν*^-^ are obtained, as shown below:


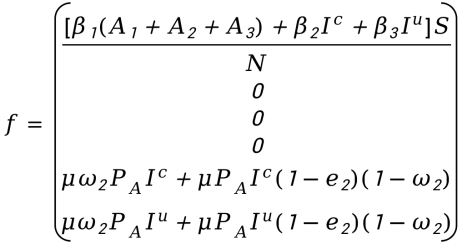


1. Matrix *f* represents the rate of new infections resulting from horizontal transmission and mother-to-child transmission. In addition, the matrix *ν* is calculated from the matrix *ν*^+^ which represents the increment rate of entering each infectious compartment and the matrix *ν*^-^ which represents the loss rate of moving out of each infectious compartment. The detailed process is shown as follows:


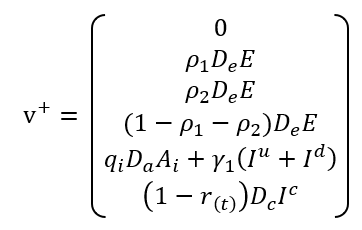

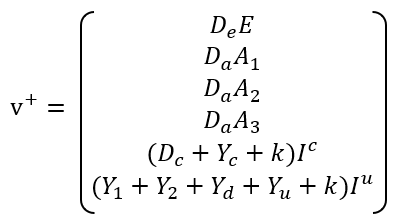

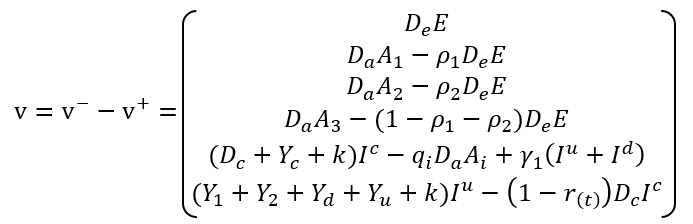


1. Calculate the Jacobian matrix of matrix *f* and *ν* respectively, that is, calculate the partial derivatives of matrix *f* and matrix *ν* with respect to the above six infectious state variables respectively, and get two new matrices F and V of 6×6, the results are as follows:


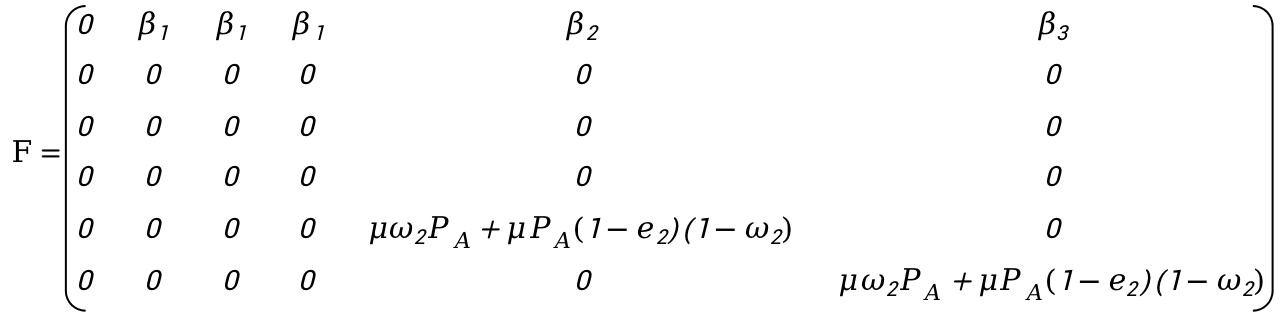

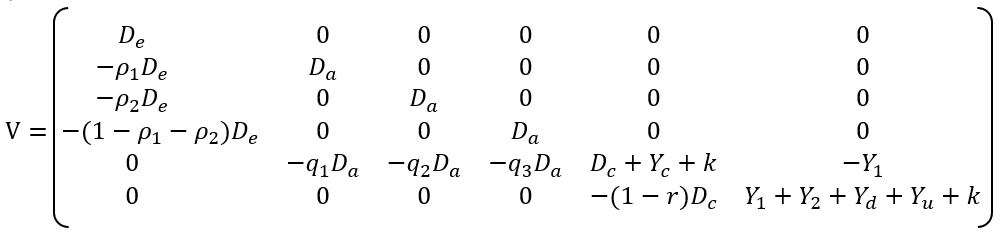


1.
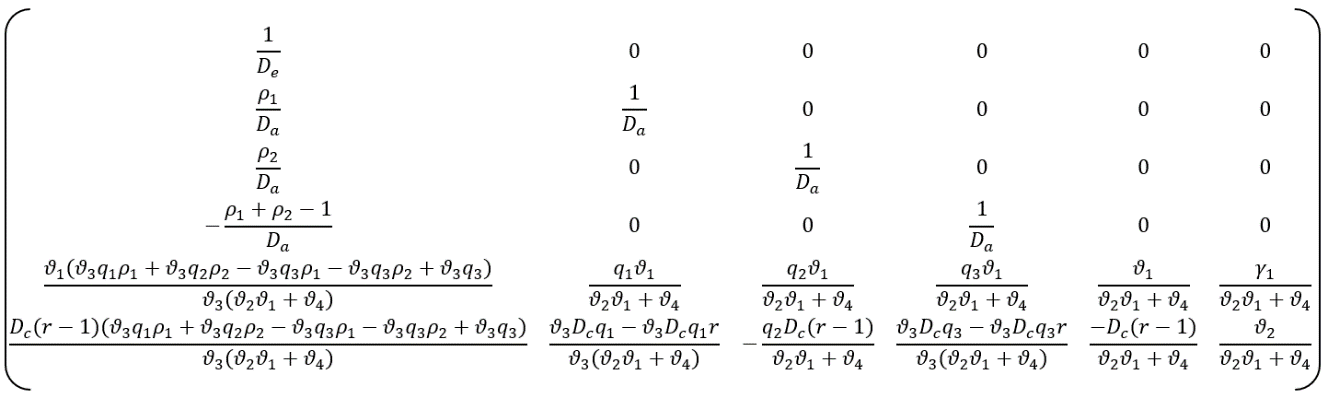
We obtained its inverse matrix V^-1^ base on the matrix V. To simplify the expression of result, let $\vartheta_{1}=Y_{1}+Y_{2}+Y_{d}+Y_{u}+k,\vartheta_{2}=D_{c}+Y_{c}+k, \vartheta_{3}= D_{a}^{3}D_{e}, \vartheta_{4}=D_{c}\left( r-1 \right)Y_{1}$, the matrix V-1 of be as follows:

In conclusion, $R_{e}=\rho\left( FV^{-1} \right)=max\left| \lambda_{i} \right|, when\left| \lambda_{i}\left( FV^{-1} \right)-E \right|=0.353.$

**3.3 Potential immunization strategies**

At present, both the infant coverage rate and the timely birth-dose vaccination rate in China have nearly reached the level of 100%. However, due to the high burden of chronic HBV infection in China, feasible immunization strategies should be further explored on the basis of maintaining the high coverage rates. In China, hepatitis B vaccine is recommended, but not mandatory, for non-infants. In our model, we simulated that in the range of coverage rate for non-infants (40%-80%), the total number of chronic HBV infections only declined with 12,967-1,444 (**Fig S5-A**); the results exhibited that the greater input in non-infant vaccination, the lower the decrease in chronic HBV infections. It’s shown that the strategy has no significant effect on any of the four outcome indicators in **Fig S5**. With each 18% increase in the parameter θ (representing an increase of magnitude of enhancing PMTCT strategy), it will decline with 17,1648 chronic HBV infections; the number of chronic HBV infections that less than 5 years old only has extra decrease of 23,952 chronic HBV infections. The average increase in the number of individuals who could avoid infection with HBV was as high as 2.08 million (**Fig S5**). In addition, the results of sensitivity analysis are shown in **Fig S6-S7**.

**3.4 Sensitivity Analysis**

This study was more intuitively understood the impact of the parameters on R_e_ and the uncertainty of the model through sensitivity analysis. Based on the derivation process and results of R_e_, it is found that the main parameters affecting R_e_ are β_2_, β_3_, D_C_, E_2_, θ and ω_2_, respectively. As can be seen from **Fig S6**, ω_2_ is the most sensitive parameter to R_e_, followed by D_c_, E_2_ and θ, while β_2_ and β_3_ are less sensitive. On the basis of maintaining the PMTCT efficiency (e_2_) as high as 99%, R_e_ decreases with the increase of the timely birth-dose vaccination rate (1-ω_2_). When the timely birth-dose vaccination rate is continuously stable at 96% or above (the current situation), R_e_ can be stable at less than 0.5. However, if the timely birth-dose vaccination rate is lower than 70%, R_e_ will exceed 1 (disease-free equilibrium threshold), leading to the outbreak of hepatitis B (**Fig S6-C**).

In addition, even if on the basis of the timely birth-dose vaccination rate is close to 100% and e_2_ is 99%, if the antiviral treatment are not implemented for women of childbearing age, the coverage rate of antiviral treatment among childbearing-age patients with chronic HBV infection in the population (θ) reaches the level of the scenario “Without the NIP”, and the R_e_ increases to 1.5 ~ 2.5. The timely birth-dose vaccination rate can be appropriately reduced only under the premise that the infectious proportion of childbearing-age women in the infectious population is reduced to the same as that of childbearing-age women in the general population and the PMTCT strategy (timely birth-dose vaccination + immunoglobulin vaccination + antiviral treatment of among childbearing-age patients with chronic HBV infection) is fully implemented (**Fig S6-A**).

Compared with the effect of immunization strategy, if the progression rate from chronic HBV infections to CHB stage can be slowed down (*e.g.*, developing more effective antiviral drugs) to prolong the carrying status of chronic HBV infectious (reduced D_c_), it will play a great role in prevent and control the hepatitis B epidemic, despite of there is a large number of chronic HBV infections in the population (**Fig S6-B**). As can be seen from **Fig S6-D**, β_2_ and β_3_ have little effect on R_e_.

The dynamic model was then used to explore the effects of the changes of β_2_, β_3_, D_c_ and e_2_ on the number of real-time chronic HBV infections (I^c^ + I^u^ + I^d^) in a certain interval above and below the set baseline values(**Fig S7**). Note: sensitivity analysis of these parameters is not considered because natural birth and death rates vary relatively steady in reality, and the progression rates of the natural course are more closely related to the disease itself. The effect of parameter θ variation on the total number of real-time chronic HBV infections within a defined interval has been described in Results of manuscript.

The analysis results showed that the progression rate (D_c_) from chronic HBV carriers to CHB infection was the most sensitive. When the baseline level increased to 0.05 and 0.1, chronic HBV infections decreased by 13.76 million and 27.38 million cases, respectively. When baseline levels dropped to 0.0025, chronic HBV infections increased by a maximum of 25.96 million, respectively. Second sensitive parameter is maternal and child block the efficient (e_2_), sensitivity analysis shows that if only the implementation of timely birth-dose vaccination strategies and without timely vaccination immunoglobulin (the effective rate of PMTCT strategy was 87.8%), it will achieve maximum growth of 4.86 million cases of chronic HBV infections; and continue to reduce to 70% of efficiency, it will cause a much more cases, about 11.7 million cases.

In addition, it’s known from the calculation principle that the influence factors of β_2_ and β_3_ may be the population contact matrix and the infectivity of HBV itself, so these parameters generally don’t fluctuate over a wide range of areas. Sensitivity analysis found that β_2_ had a greater effect on the number of chronic HBV infections than β_3_, with an average increase of 3.81 million and 770 million chronic HBV infections when the baseline levels of the two parameters were increased by 10 times, respectively.

Sensitivity analysis of the two parts has drawn relatively consistent conclusions.

**Table S1. Critical parameters of HBV mathematical model**

| Parameter | Description | Value | Source |
| --- | --- | --- | --- |
| μ(t) | Birth rate in year t | See **Table S3** | National Bureau of Statistics |
| k(t) | Death rate in year t | See **Table S3** | National Bureau of Statistics |
| ω_1_ | 1- (infant vaccination coverage in year t) | See **Table S3** | [5, 6] |
| ω_2_ | 1- (timely birth-dose vaccination coverage in year t) | See **Table S3** | [5, 6] |
| b | The proportion of women HBV infections (15-49 years) in chronic HBV infections/the proportion of women individuals (15-49 years) in general population | 2.2  (Calculated based on survey data in 1992) | [7] |
| θ | The coverage rate of antiviral treatment among childbearing-age patients with chronic HBV infection | 0%-72% | Assumed |
| β_1_ | Transmission coefficient of the total acute infections | 0.026 | Least square method |
| β_2_ | Transmission coefficient of chronic HBV infections | 0.030 | Least square method |
| β_3_ | Transmission coefficient of chronic hepatitis B | 0.021 | Least square method |
| h | Latent period | 6 | [8] |
| s | Duration of acute infectious period | 4 | [8] |
| p | Vaccination coverage rate of non-infant individuals | 0-0.3 | Assumed |
| $\rho_{1}$ | The proportion of acute infections under 1 year of age | 0.005 | GBD[9] |
| $\rho_{2}$ | The proportion of acute infections under 1-5 year of age | 0.02 | GBD[9] |
| q_1_ | Proportion of acute HBV infections (< 1 years) become chronic infectiousness | 0.9 | [10] |
| q_2_ | Proportion of acute HBV infections (1-5 years) become chronic infectiousness | 0.275 | [10] |
| q_3_ | Proportion of acute HBV infections (>5 years) become chronic infectiousness | 0.075 | [10] |
| $\varphi$ | Loss rate of HBV vaccine effectiveness | 0.05 | Guidelines for the prevention and treatment of chronic hepatitis B (2015 edition) |
| D_c_ | Rate of chronic HBV infections becoming CHB | 0.025 | Assumed |
| γ_1_ | Rate of CHB becoming chronic HBV infections | 0.00608 | [11] |
| γ_2_ | Recovery rate of CHB | 0.025 | [8] |
| γ_3_ | Rate of CC becoming HCC | 0.03 | Guidelines for the prevention and treatment of chronic hepatitis B (2015 edition) |
| γ_c_ | Rate of chronic HBV infections becoming HCC | 0.005 | [12] |
| γ_u_ | Rate of transition from CHB becoming HCC | 0.004 | Guidelines for the prevention and treatment of chronic hepatitis B (2015 edition) |
| e_1_ | Efficacy of infant vaccination | 0.90 | [13] |
| e_2_ | Efficacy of timely birth-dose vaccination | 0.878-0.990 | [13] |
| e_3_ | Efficacy of HBV vaccination in non-infant | 0.70 | [13] |

**Table S2. Parameters of decision tree-Markov model**

| Parameter | Value | Range |
| --- | --- | --- |
| Outcome probabilities of acute infection |  |  |
| Symptomatic[11, 14] |  |  |
| <1 year | 0.01 | ±50% |
| 1-10 years | 0.1 |  |
| ≥11 years | 0.3 |  |
| Symptomatic infections that require hospitalization[15] | 0.12 | 0.01-0.5 |
| Hospitalized cases that are fulminant[16] | 0.04 | 0.01-0.1 |
| Fulminant cases that result in death[15] | 0.65 | 0.4-0.8 |
| Acute infections that become chronic[17] |  |  |
| <1 year | 0.3 | ±50% |
| 1-5 years | 0.25 |  |
| 6-19 years | 0.06 |  |
| ≥20 years | 0.04 |  |
| Annual transition probabilities related to chronic infection |  |  |
| Asymptomatic carrier |  |  |
| To immune[18] | 0.012 | ±50% |
| To chronic hepatitis[19] |  |  |
| 0-19 years | 0.0012 |  |
| 20-39 years | 0.0023 |  |
| ≥40 years | 0.0054 |  |
| To hepatocellular carcinoma[19] |  |  |
| 0-19 years | 0.0005 | ±50% |
| 20-39 years | 0.002 |  |
| ≥40 years | 0.0061 |  |
| Chronic hepatitis |  |  |
| To Asymptomatic carrier[20] | 0.00608 | 0-0.05 |
| To compensated cirrhosis[17] |  |  |
| 0-19 years | 0.0081 | ±50% |
| 20-39 years | 0.02 |  |
| ≥40 years | 0.027 |  |
| To hepatocellular carcinoma[21] |  |  |
| 0-19 years | 0.003 | ±50% |
| 20-39 years | 0.005 |  |
| ≥40 years | 0.0064 |  |
| Compensated cirrhosis |  |  |
| To Decompensated cirrhosis[22] | 0.07 | 0.03-0.1 |
| To hepatocellular carcinoma[22] | 0.034 | 0.01-0.1 |
| Decompensated cirrhosis |  |  |
| To hepatocellular carcinoma[22] | 0.034 | 0.01-0.1 |
| To death[22] | 0.144 | 0.1-0.25 |
| Hepatocellular carcinoma |  |  |
| To death[22] | 0.5 | 0.3-0.7 |
| HBsAg screening | 0.965 | 0.8-0.974 |
| HBsAg positive women | 0.069 | 0.061-0.077 |
| HBIG coverage | 0.888 | 0.85-0.974 |
| Costs[23] |  |  |
| Acute infection in hospital | 2438.84 | ±50% |
| Acute infection not in hospital | 118.51 |  |
| Chronic infection | 3395.14 |  |
| Compensated cirrhosis | 5359.84 |  |
| Decompensated cirrhosis | 6956.43 |  |
| Hepatocellular carcinoma | 11128.66 |  |
| HBV vaccine | 3.18 | 16-25 |
| HBIG | 22.99 | 145-176 |
| Screening | 1.21 | 6-10 |
| Utility scores[22, 24] |  |  |
| Acute symptomatic infection |  |  |
| Not fulminant | 0.9 | 0.8-1 |
| Fulminant | 0.73 | 0.5-0.8 |
| Chronic hepatitis | 0.789 | 0.7-1 |
| Compensated cirrhosis | 0.763 | 0.7-0.9 |
| Decompensated cirrhosis | 0.661 | 0.5-0.8 |
| Hepatocellular carcinoma | 0.699 | 0.5-0.8 |
| Discount rate | 0.03 | 0-0.05 |

Note: All costs were adjusted with respect to the cost of dollar according to the exchange rate in 2018.

**Table S3. Time-varied data of birth, death and vaccination rates**

| Year | Infant vaccination rate | Timely birth-dose vaccination rate | Birth rate | Death rate |
| --- | --- | --- | --- | --- |
| 1990 | 0 | 0 | 0.02106 | 0.00667 |
| 1991 | 0 | 0 | 0.01968 | 0.00670 |
| 1992 | 0.300 | 0.222 | 0.01824 | 0.00664 |
| 1993 | 0.343 | 0.237 | 0.01809 | 0.00664 |
| 1994 | 0.381 | 0.225 | 0.01770 | 0.00649 |
| 1995 | 0.397 | 0.271 | 0.01712 | 0.00657 |
| 1996 | 0.452 | 0.282 | 0.01698 | 0.00656 |
| 1997 | 0.559 | 0.394 | 0.01657 | 0.00651 |
| 1998 | 0.568 | 0.391 | 0.01603 | 0.00650 |
| 1999 | 0.612 | 0.437 | 0.01464 | 0.00646 |
| 2000 | 0.766 | 0.541 | 0.01403 | 0.00645 |
| 2001 | 0.830 | 0.644 | 0.01338 | 0.00643 |
| 2002 | 0.843 | 0.661 | 0.01286 | 0.00641 |
| 2003 | 0.877 | 0.689 | 0.01241 | 0.00640 |
| 2004 | 0.900 | 0.760 | 0.01229 | 0.00642 |
| 2005 | 0.840 | 0.860 | 0.01240 | 0.00651 |
| 2006 | 0.910 | 0.880 | 0.01209 | 0.00681 |
| 2007 | 0.920 | 0.860 | 0.01210 | 0.00693 |
| 2008 | 0.950 | 0.910 | 0.01214 | 0.00706 |
| 2009 | 0.991 | 0.930 | 0.01195 | 0.00708 |
| 2010 | 0.995 | 0.920 | 0.01190 | 0.00711 |
| 2011 | 0.996 | 0.950 | 0.01193 | 0.00714 |
| 2012 | 0.997 | 0.960 | 0.01210 | 0.00715 |
| 2013 | 0.996 | 0.990 | 0.01208 | 0.00716 |
| 2014 | 0.994 | 0.940 | 0.01237 | 0.00716 |
| 2015 | 0.996 | 0.960 | 0.01207 | 0.00711 |
| 2016 | 0.995 | 0.960 | 0.01295 | 0.00709 |
| 2017 | 0.995 | 0.960 | 0.01243 | 0.00711 |
| 2018 | 0.995 | 0.960 | 0.01094 | 0.00713 |


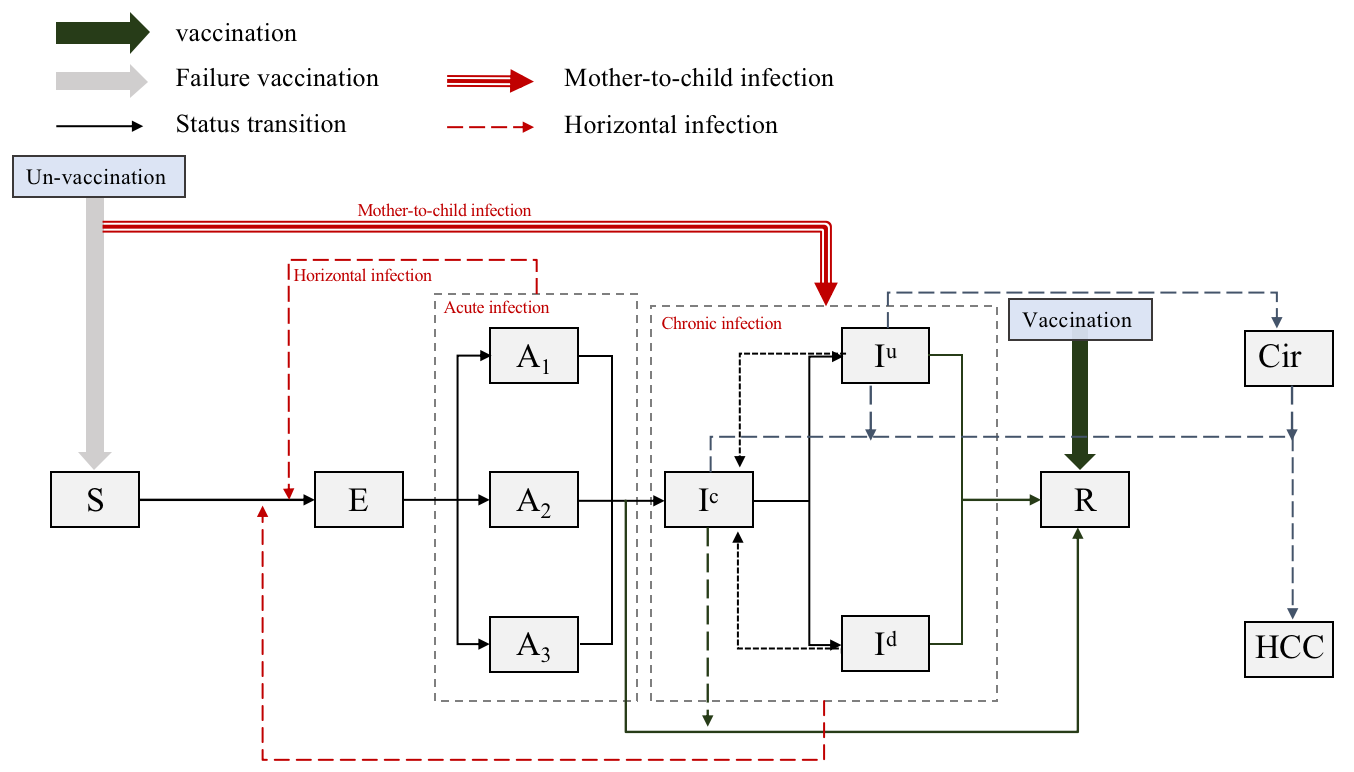


**Fig S1**. Transmission structure of the HBV mathematical model


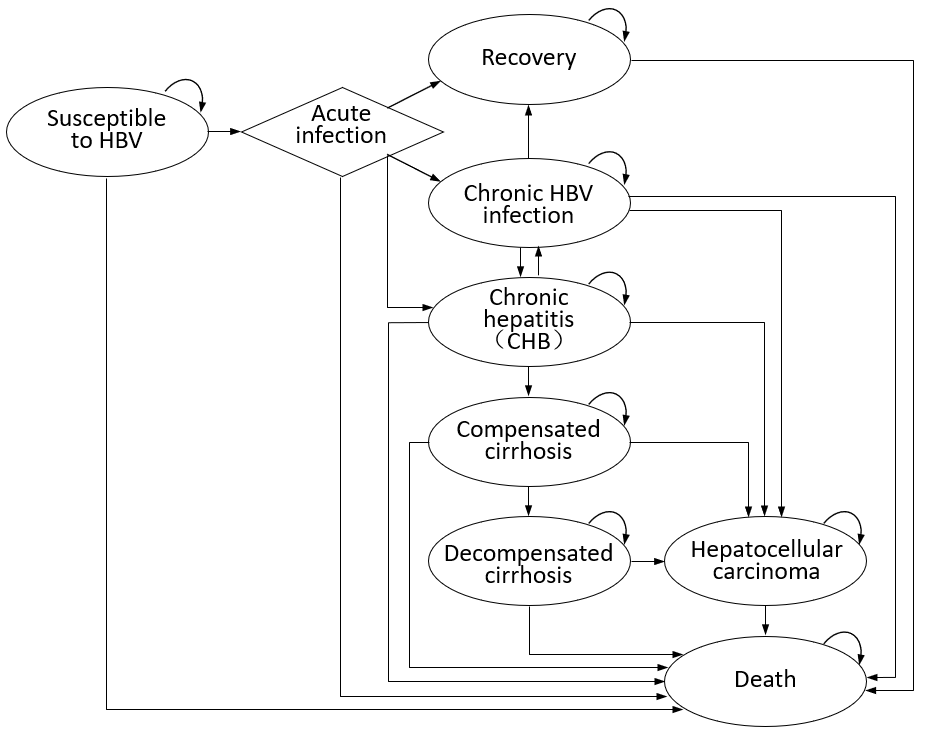


**Fig S2. HBV infection and progression structure of Markov model**


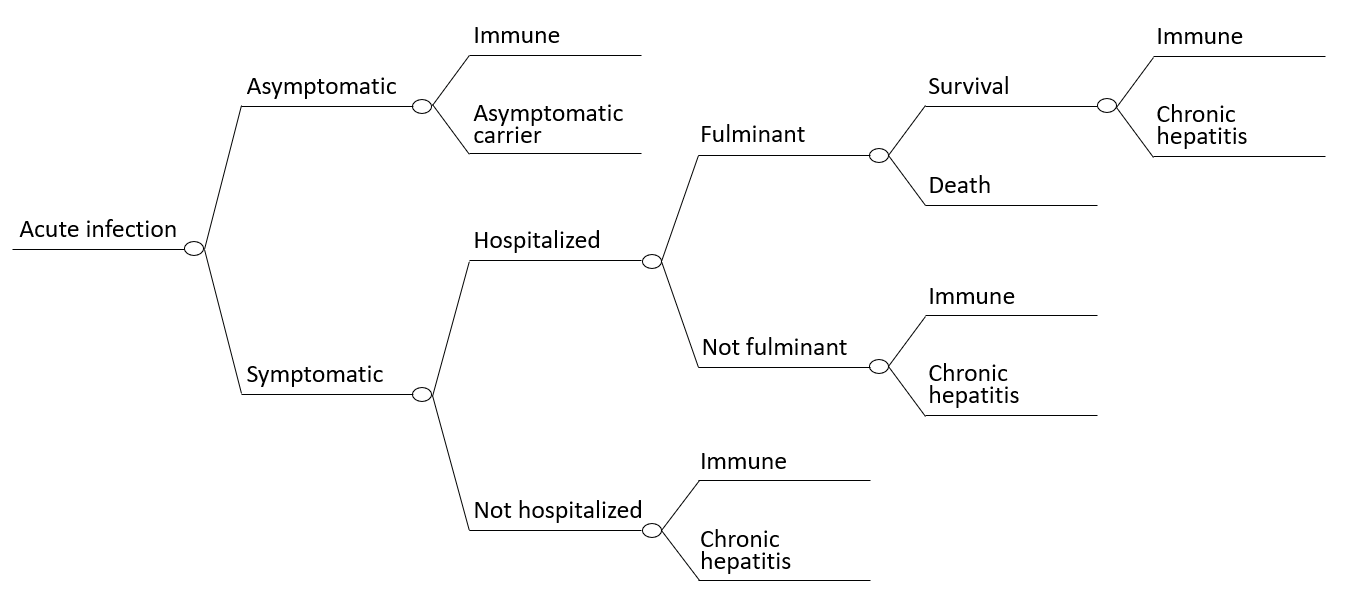


**Fig S3. Process of acute HBV infection**


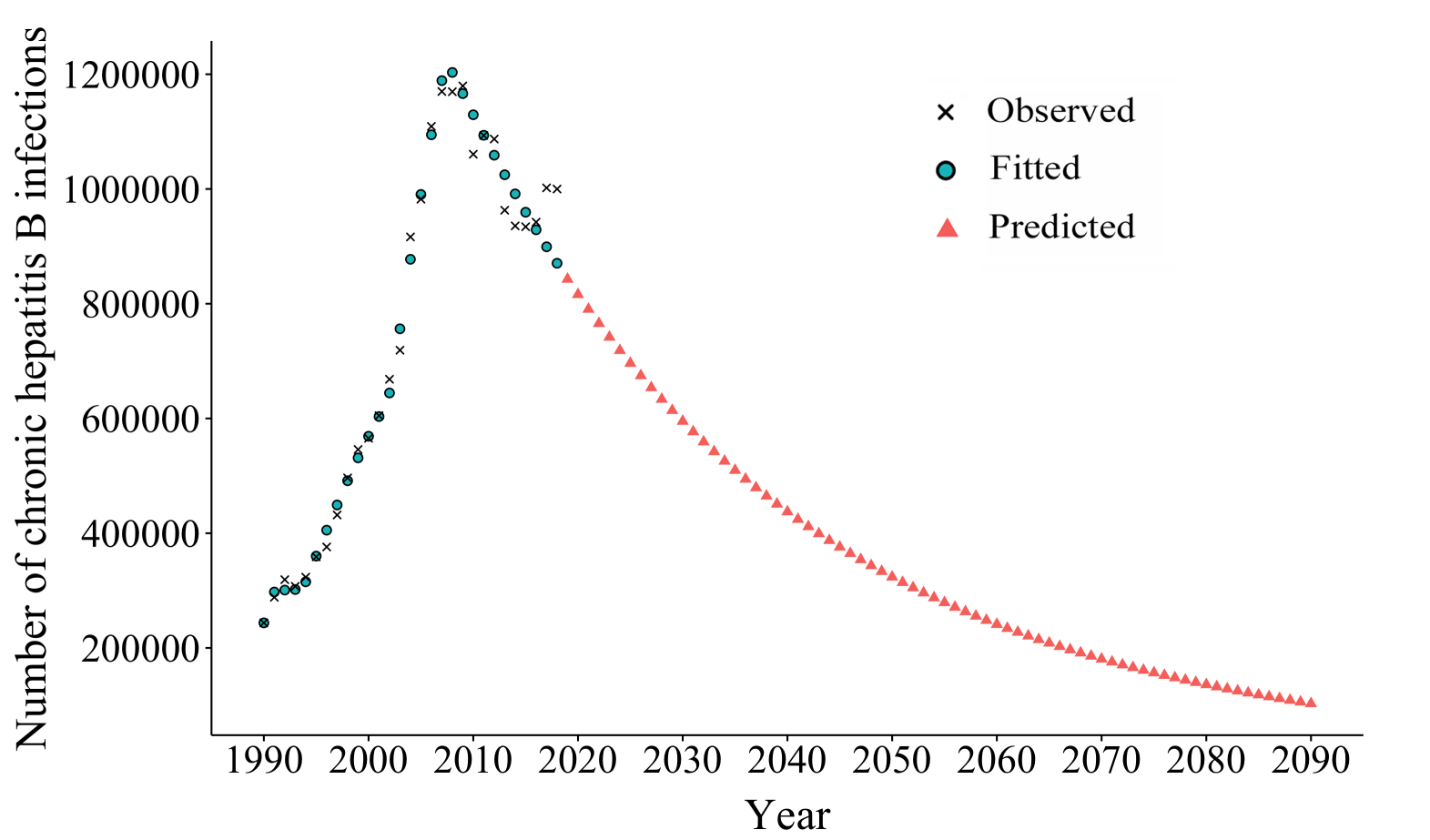


**Fig S4. Dynamic model fitting and prediction results**

The blue points represent the observed incidence data, while the black cross represents the incidence data fitted by the model. Red triangles represent the predicted incidence data.


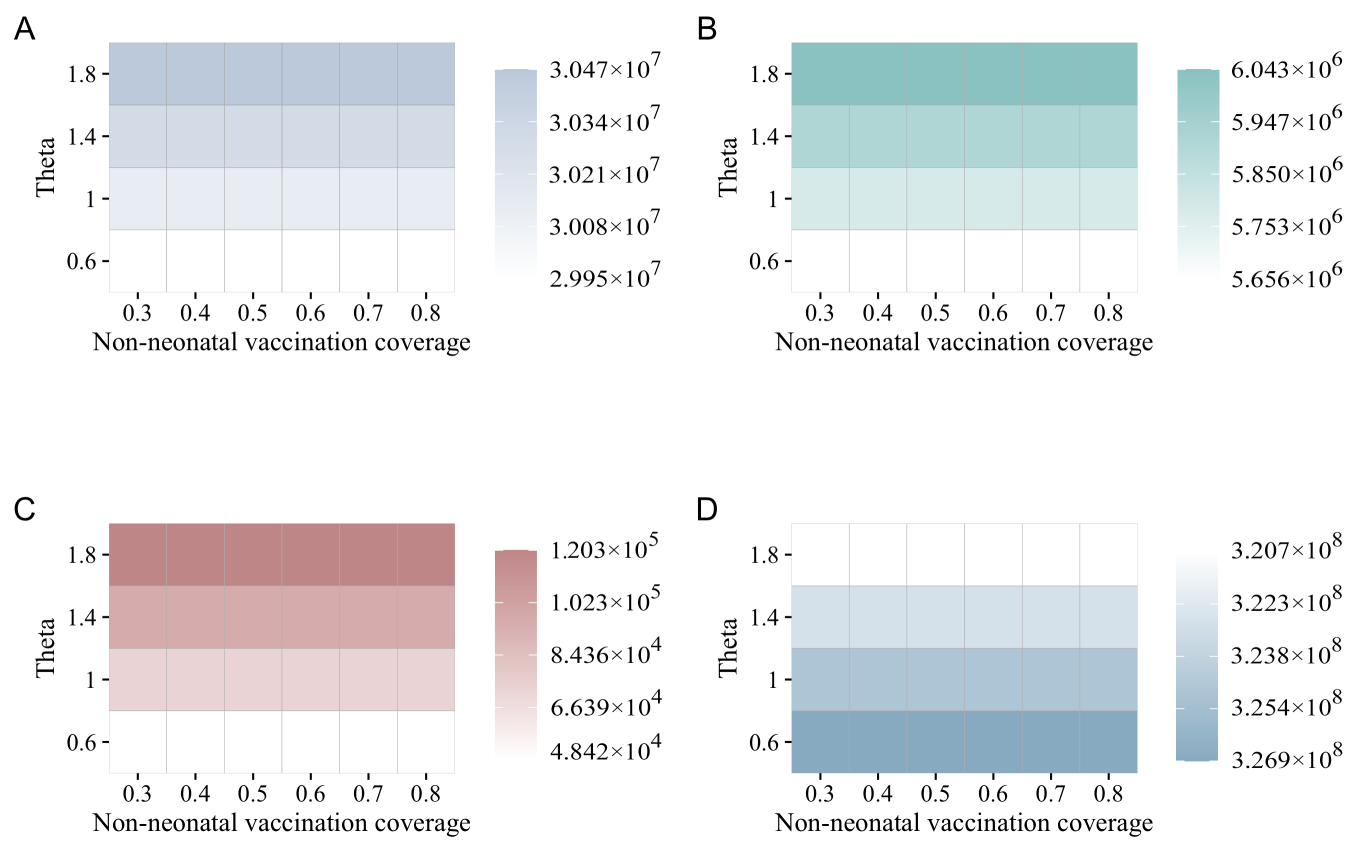


The total number of chronic

infectious cases

The number of MTCT cases

The number of chronic

infectious cases (≤5 years old)

The number of

averted infectious cases

**Fig S5. Exploration of feasible vaccination interventions on hepatitis B.**

A: the impact of varied coverage rates of antiviral treatment in childbearing-age patients with chronic HBV infection and non-infant vaccination rates on the total chronic HBV infections; B: the impact of varied coverage rates of antiviral treatment in childbearing-age patients with chronic HBV infection and non-infant vaccination rate on the MTCT cases; C: the impact of varied coverage rates of antiviral treatment in childbearing-age patients with chronic HBV infection and non-infant vaccination rates on the chronic HBV infections under five years old; D: the impact of varied coverage rates of antiviral treatment in childbearing-age patients with chronic HBV infection and non-infant vaccination rates on the number of people who avoided HBV infection. MTCT: mother-to-child transmission.


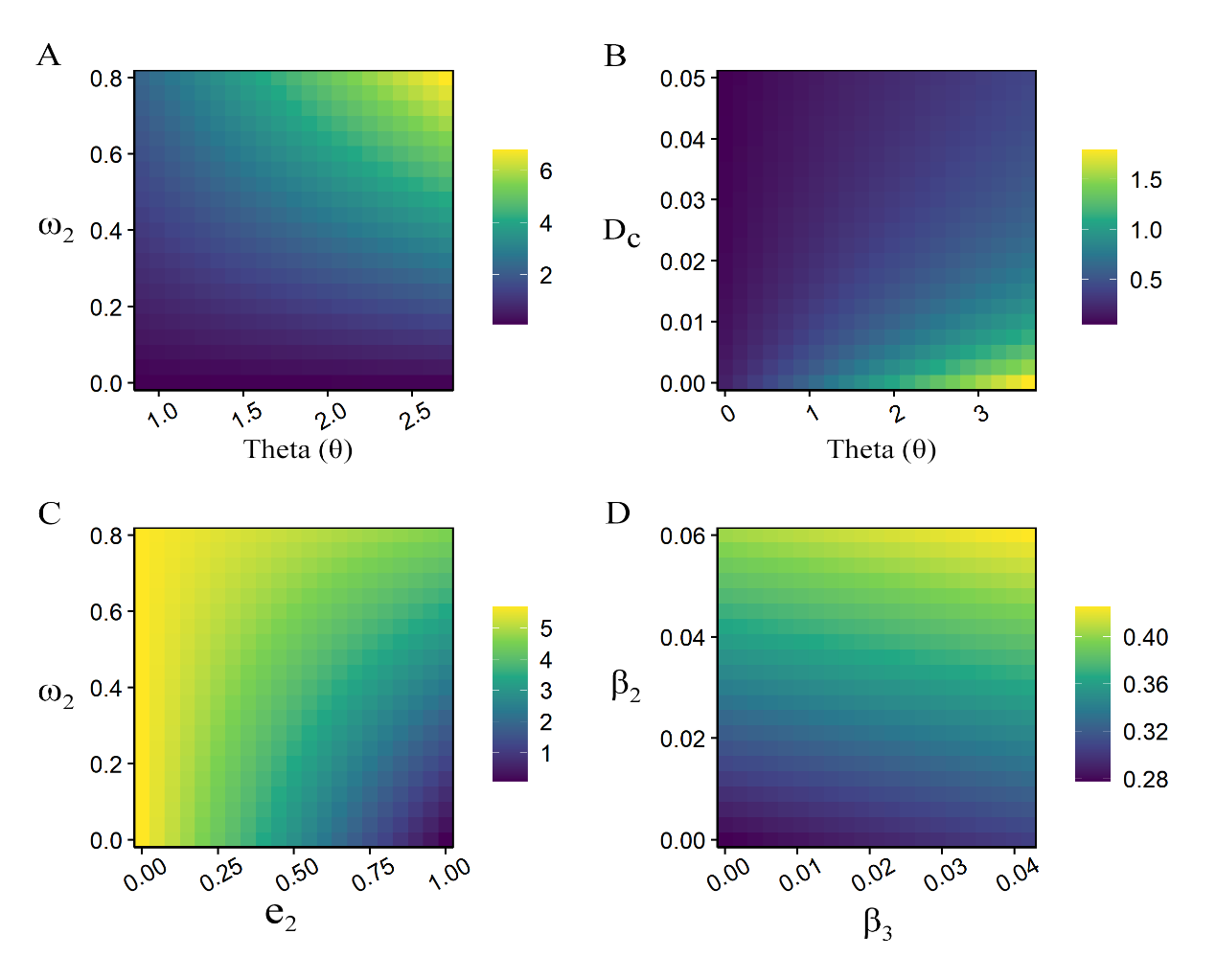


**Fig S6. Sensitivity analysis of parameters and R_e_**

Estimated impacts of parameters on the R_e_, with varied ranges based on sensitivity analysis. A, B, C, D show the parameters (β_2_, β_3_, D_c_, e_2_, $\theta, \omega_{2}$) range below to above of their setting values, respectively.


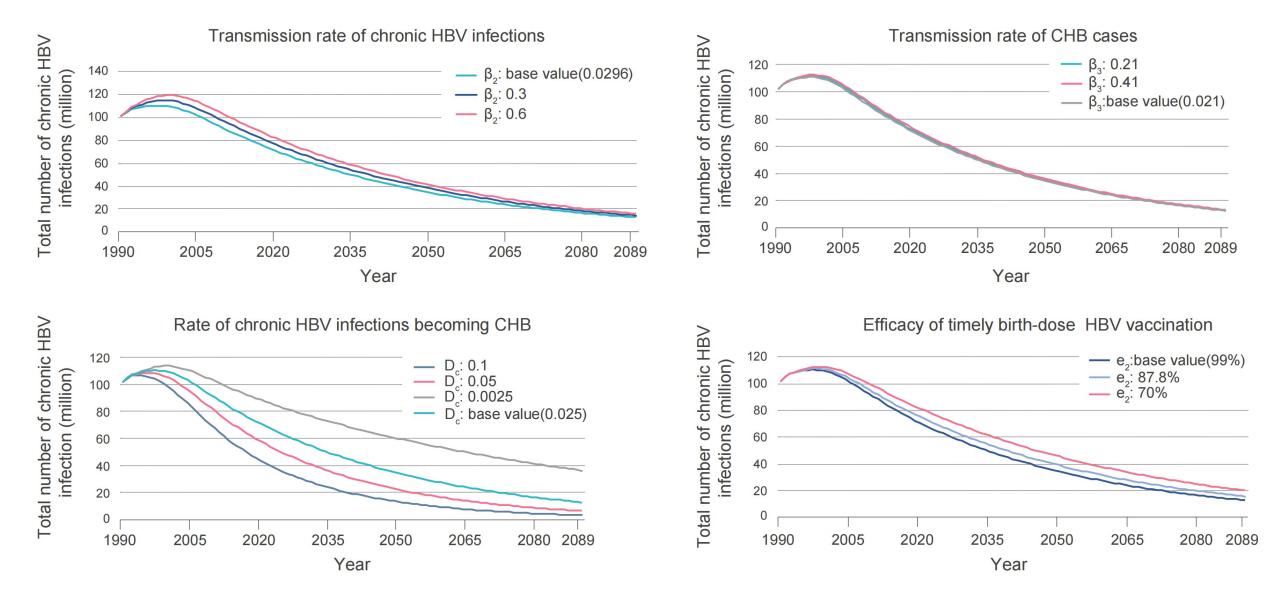


**Fig S7. Sensitivity analysis results of parameters**

Estimated impacts of parameters on the number of chronic HBV infections (I^c^+I^u^+I^d^), with varied ranges based on sensitivity analysis. A, B, C, D show the four parameters (β_2_, β_3_, D_c_, e_2_) range below to above of their setting values, respectively.


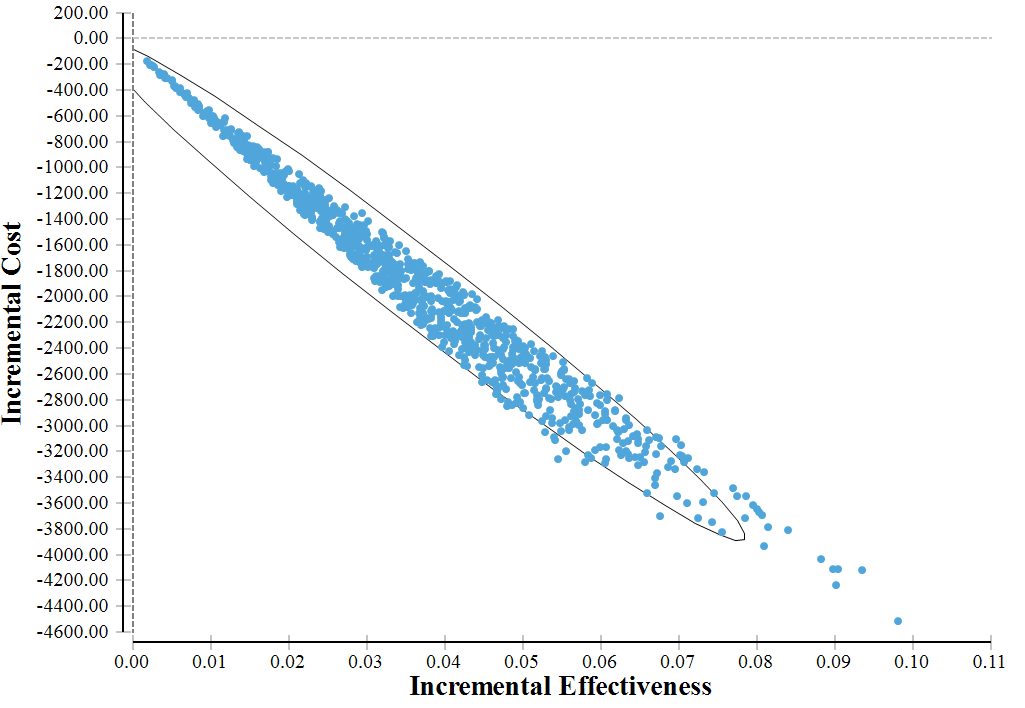


**Fig S8. Results of Probabilistic Sensitivity Analysis**

Incremental Cost-Effectiveness among the PMTCT vs. Infant vaccination strategy (conducted in “Status quo” scenario).

**Reference**

1. Edmunds WJ, Medley GF, Nokes DJ, Hall AJ, Whittle HC. The influence of age on the development of the hepatitis B carrier state. Proc Biol Sci. 1993;253(1337):197-201.

2. Trepo C, Chan HLY, Lok A. Hepatitis B virus infection. Lancet (London, England). 2014;384(9959):2053-63.

3. Diekmann O, Heesterbeek JA, Metz JA. On the definition and the computation of the basic reproduction ratio R0 in models for infectious diseases in heterogeneous populations. J Math Biol. 1990;28(4):365-82.

4. van den Driessche P, Watmough J. Reproduction numbers and sub-threshold endemic equilibria for compartmental models of disease transmission. Math Biosci. 2002;180:29-48.

5. WHO. Hepatitis B immunization coverage among 1-year-olds. World Health Organization. 2021.

6. Liang X, Bi S, Yang W, Wang L, Cui G, Cui F, et al. Evaluation of the Impact of Hepatitis B Vaccination among Children Born during 1992–2005 in China. The Journal of Infectious Diseases. 2009;200(1):39-47.

7. Xia G-L, Liu C-B, Cao H-L, Bi S-L, Zhan M-Y, Su C-A, et al. Prevalence of hepatitis B and C virus infections in the general Chinese population. Results from a nationwide cross-sectional seroepidemiologic study of hepatitis A, B, C, D, and E virus infections in China, 1992. International Hepatology Communications. 1996;5(1):62-73.

8. Shepard CW, Simard EP, Finelli L, Fiore AE, Bell BP. Hepatitis B virus infection: epidemiology and vaccination. Epidemiol Rev. 2006;28:112-25.

9. GBD. GBD Results Tool. Global Burden of Disease. 2021.

10. Lai CL, Ratziu V, Yuen MF, Poynard T. Viral hepatitis B. Lancet (London, England). 2003;362(9401):2089-94.

11. Yin J, Ji Z, Liang P, Wu Q, Cui F, Wang F, et al. The doses of 10 μg should replace the doses of 5 μg in newborn hepatitis B vaccination in China: A cost-effectiveness analysis. Vaccine. 2015;33(31):3731-8.

12. Lin X, Robinson NJ, Thursz M, Rosenberg DM, Weild A, Pimenta JM, et al. Chronic hepatitis B virus infection in the Asia-Pacific region and Africa: review of disease progression. J Gastroenterol Hepatol. 2005;20(6):833-43.

13. Xia GL GJ, Wang JJ, Meng ZD, Jia ZY, Cao HL, Liu CB. Efficacy of recombinant hepatitis B vaccine and low—dose hepatitis B immune globulin in preventing mother-infant transmission of hepatitis B virus infection. Chinese Journal of Epidemiology. 2003(05):32-5.

14. Fabrizi F, Martin P. Hepatitis B virus infection in dialysis patients. American journal of nephrology. 2000;20(1):1-11.

15. Trépo C, Chan HL, Lok A. Hepatitis B virus infection. (1474-547X (Electronic)).

16. Gibson E, Begum N, Sigmundsson B, Sackeyfio A, Hackett J, Rajaram S. Economic evaluation of pediatric influenza immunization program compared with other pediatric immunization programs: A systematic review. Human vaccines & immunotherapeutics. 2016;12(5):1202-16.

17. Busch K, Thimme R. Natural history of chronic hepatitis B virus infection. (1432-1831 (Electronic)).

18. Chu CM, Liaw YF. HBsAg seroclearance in asymptomatic carriers of high endemic areas: appreciably high rates during a long-term follow-up. (0270-9139 (Print)).

19. Hung HF, Chen TH. Probabilistic cost-effectiveness analysis of the long-term effect of universal hepatitis B vaccination: an experience from Taiwan with high hepatitis B virus infection and Hepatitis B e Antigen positive prevalence. (1873-2518 (Electronic)).

20. Zheng H, Wang FZ, Zhang GM, Cui FQ, Wu ZH, Miao N, et al. An economic analysis of adult hepatitis B vaccination in China. Vaccine. 2015;33(48):6831-9.

21. Chen YS, Zheng H, Liu YM, Wang FZ, Wu ZH, Miao N, et al. Economic evaluation on infant hepatitis B vaccination combined with immunoglobulin in China, 2013. Human vaccines & immunotherapeutics. 2016;12(7):1838-46.

22. Jia Y, Li L, Cui F, Zhang D, Zhang G, Wang F, et al. Cost-effectiveness analysis of a hepatitis B vaccination catch-up program among children in Shandong Province, China. Human vaccines & immunotherapeutics. 2014;10(10):2983-91.

23. Zhang S, Ma Q, Liang S, Xiao H, Zhuang G, Zou Y, et al. Annual economic burden of hepatitis B virus-related diseases among hospitalized patients in twelve cities in China. (1365-2893 (Electronic)).

24. La Torre G, Mannocci A, Saulle R, Colamesta V, Meggiolaro A, Mipatrini D, et al. Economic evaluation of HBV vaccination: A systematic review of recent publications (2000-2013). Human vaccines & immunotherapeutics. 2016;12(9):2299-311.
